# Virtual-scanning light-field microscopy for robust snapshot high-resolution volumetric imaging

**DOI:** 10.1038/s41592-023-01839-6

**Published:** 2023-04-06

**Authors:** Zhi Lu, Yu Liu, Manchang Jin, Xin Luo, Huanjing Yue, Zian Wang, Siqing Zuo, Yunmin Zeng, Jiaqi Fan, Yanwei Pang, Jiamin Wu, Jingyu Yang, Qionghai Dai

**Affiliations:** 1 Department of Automation, Tsinghua University, Beijing, China; 2 Institute for Brain and Cognitive Sciences, Tsinghua University, Beijing, China; 3 School of Electrical and Information Engineering, Tianjin University, Tianjin, China; 4 IDG/McGovern Institute for Brain Research, Tsinghua University, Beijing, China

**Keywords:** Microscopy, Fluorescence imaging

## Abstract

High-speed three-dimensional (3D) intravital imaging in animals is useful for studying transient subcellular interactions and functions in health and disease. Light-field microscopy (LFM) provides a computational solution for snapshot 3D imaging with low phototoxicity but is restricted by low resolution and reconstruction artifacts induced by optical aberrations, motion and noise. Here, we propose virtual-scanning LFM (VsLFM), a physics-based deep learning framework to increase the resolution of LFM up to the diffraction limit within a snapshot. By constructing a 40 GB high-resolution scanning LFM dataset across different species, we exploit physical priors between phase-correlated angular views to address the frequency aliasing problem. This enables us to bypass hardware scanning and associated motion artifacts. Here, we show that VsLFM achieves ultrafast 3D imaging of diverse processes such as the beating heart in embryonic zebrafish, voltage activity in *Drosophila* brains and neutrophil migration in the mouse liver at up to 500 volumes per second.

## Abstract (web summary)

Virtual-scanning light-field microscopy (VsLFM) uses a physics-based deep learning model to improve the quality and speed of LFM, reducing motion artifacts and enabling challenging demonstrations such as fast 3D voltage imaging in *Drosophila*.

## Main

Understanding the interaction and function between multiple cells and organelles in living organisms requires high-resolution robust volumetric imaging at high speed. In the past decade, various efforts in three-dimensional (3D) fluorescence imaging have been made to promote the rapid development of cell biology^1–6^, developmental biology^7–10^ and neuroscience^11–16^. Of these, light-field microscopy (LFM) has been widely used in neural recordings of diverse animals with cellular resolution, due to its compact optical system and snapshot volumetric imaging capability^15–19^. By simultaneously exciting and collecting all fluorescence photons from the entire volume, LFM facilitates long-term high-speed intravital imaging in mammals at low phototoxicity^20^. Although numerous reconstruction algorithms have been developed to enable the practical and versatile application of LFM in biology^21–27^, LFM is still hindered by low spatial resolution and reconstruction artifacts, especially in complicated intravital environments. By introducing periodic beam drifting to increase the spatial sampling density, scanning LFM (sLFM) increases the resolution up to the diffraction limit and facilitates multi-site aberration correction in post-processing^28^, but the physical scanning process reduces the 3D imaging speed and may introduce motion artifacts for highly dynamic samples^20^.

Meanwhile, with the rapid development of deep learning, many emerging learning-based algorithms have been introduced in LFM^29–33^ to improve the reconstruction speed and resolution using light-sheet microscopy^32^ or confocal microscopy^31,33^ as the ground truth. However, there are still three main bottlenecks for these end-to-end networks. First, by mapping the low-resolution multi-view data to high-resolution 3D volumes directly, the spatial resolution of current learning-based LFM is far from sufficient for subcellular structures, due to the huge resolution gap between the raw light-field measurements and the diffraction limit of the objective. Second, end-to-end networks are susceptible to the model of the imaging process and face severe degradation in optically challenging environments, such as low signal-to-noise ratio and strong optical aberrations induced by tissue heterogeneity or imperfect imaging systems. Last, existing data-driven end-to-end approaches show poor generalization across a wide variety of biological samples, placing demanding requirements on the training dataset. Therefore, robust snapshot 3D imaging of subcellular dynamics remains a challenge for the study of transient biological dynamics in animals.

Here, we propose a physics-based deep neural network to increase the resolution of LFM based on an sLFM dataset by exploring the frequency aliasing between different angles, termed virtual-scanning light-field microscopy (VsLFM). Given that the low-resolution unscanned spatial–angular views could be extracted directly from the high-resolution data obtained by sLFM, we generated an open-source sLFM dataset, named Bio-LFSR, with well-matched high-resolution and low-resolution pairs across a wide range of species, structures and imaging conditions. With the physical constraint of frequency aliasing in multiple angular views induced by the diffraction of the small microlens aperture, VsLFM achieves better spatial resolution, robustness to optically challenging environments and generalizability to diverse sample structures than previous end-to-end methods. Working on the same compact system as a traditional LFM without the requirement of physical scanning, VsLFM achieved ~230 nm lateral resolution and 420 nm axial resolution across a large volume of 210 × 210 × 18 μm^3^ within a snapshot. Compared with sLFM, VsLFM eliminates motion artifacts with better temporal resolution for highly dynamic samples such as the beating heart, blood flow and voltage activities, while maintaining the capability of multi-site digital adaptive optics (DAO) and low phototoxicity. To demonstrate its unique advantages, we quantitatively validated VsLFM on both synthetic and experimental data. As demonstrations, we observed various transient 3D subcellular dynamics in cultured cells, a zebrafish embryo, zebrafish larvae, *Drosophila* and mice during different physiological processes at a camera frame rate of up to 500 volumes per second (vps).

## Results

### Principle of VsLFM

**Fig. 1:**
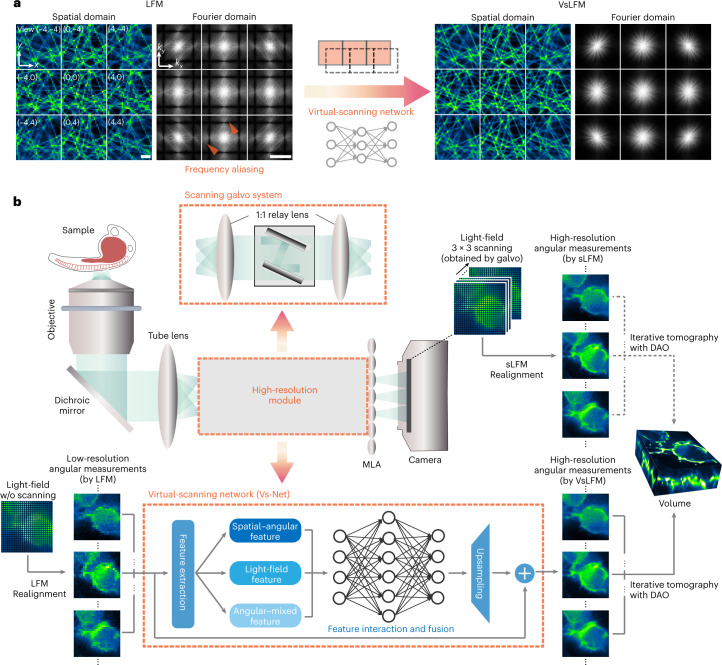
Principle of VsLFM. **a**, Principle of VsLFM using a physics-based deep neural network (Vs-Net) to extract the high-frequency information from the frequency aliasing in traditional LFM induced by diffraction of a small microlens aperture and low spatial sampling density. Such a process can be viewed as a virtual-scanning process to increase the spatial sampling density. **b**, Schematic diagram of the optical system and processing pipeline of VsLFM. In sLFM, a 2D scanning galvo shifts the image plane by 3 × 3 times physically to increase the sampling rate of angular views, which is limited by the physical size of each microlens in LFM. For a VsLFM system without a scanning galvo system, the microlens array (MLA) is placed at the back focal plane of the tube lens, and the length of the whole optical path is shortened. VsLFM uses a supervised-learning network (Vs-Net) including the extraction, interaction, fusion and upsampling of multiple spatial–angular features to realize the scanning process virtually. High-resolution angular measurements obtained by sLFM serve as the ground truth during network training to learn the physical prior between the phase-correlated low-resolution angular measurements. Finally, iterative tomography with DAO is implemented on multiple angular views obtained by Vs-Net to reconstruct 3D high-resolution volumes. Scale bars, 10 μm (spatial domain) and 1 μm^−1^ (Fourier domain) (**a**).

Our previous studies^20,28^ have shown that the microlens array inserted at the image plane can preserve the high-frequency spatial information in the low-frequency region during pupil segmentation for angular sampling due to the diffraction effect of the small microlens aperture. Such a process is akin to the structured illumination microscopy in the detection path, which ensures that the multiple angular images obtained by LFM are phase correlated, even for incoherent fluorescence light. However, the physical size of each microlens restricts the spatial sampling density, further exacerbating the frequency aliasing problem (Fig. 1a). Previous deconvolution algorithms^25,34^ use cubic interpolation during reconstruction, which ignores the effect of frequency aliasing and results in grid-like artifacts and low resolution (Supplementary Fig. 1). By shifting the light field periodically, sLFM uses physical scanning to increase the spatial sampling density (Supplementary Fig. 1). Only 3 × 3 scanning number is sufficient to address the frequency aliasing problem and recover the resolution up to the diffraction limit of the whole-objective numerical aperture (NA) for the light-field system with 13 × 13 angular views. However, such a physical scanning process will reduce the temporal resolution and introduce motion artifacts if there are strong morphological or intensity changes in the samples during the scanning process of 9 camera frames.

We have therefore developed a virtual-scanning network (Vs-Net) in the spatial–angular domain to replace the physical scanning process in sLFM for highly dynamic samples (Fig. 1a). The Vs-Net is designed to exploit the phase correlation between different angles introduced by the microlens diffraction and extract the high-frequency information from the complicated frequency aliasing by considering multiple angular measurements during the upsampling process instead of each angle separately (Fig. 1b). We use the proposed Vs-Net to map the low-resolution spatial–angular views acquired by LFM to high-resolution views, with 3 × 3 sLFM acquisitions as ground truth. Unlike end-to-end networks, which need two systems to acquire light-field images and target volumes separately, data pairs required for Vs-Net training can be simultaneously captured on the sLFM system, whereby all of the pairs are intrinsically well matched without the requirement of extra pixel registration or processing. To leverage the frequency aliasing in different spatial–angular views, we first impose a feature extractor to yield three types of features, which are then fully mixed, interacted and fused in elaborately designed modules, and are finally upsampled by a pixel shuffle to predict high-resolution spatial–angular views (Fig. 1b and Supplementary Fig. 2). With three interaction modules working collaboratively, Vs-Net can be regarded as an effective high-resolution module to replace hardware scanning in sLFM for highly dynamic samples (Fig. 1b and Supplementary Fig. 3). More details on the network architecture and size parameters are given in Methods and Supplementary Table 1. After being strengthened by Vs-Net, the spatial–angular views are fed into iterative tomography with DAO^20^, a proven and general framework independent of sample structures and imaging conditions. Point spread functions are then imposed as another physical prior to reconstruct 3D high-resolution volumes up to the diffraction limit. Our two-step physics-based learning framework can then fill the huge resolution gap, usually at a factor greater than 10, corresponding to the ratio between the resolution of raw LFM measurements and the diffraction limit of the objective, which hinders traditional LFM^15,25^ or previous end-to-end learning-based LFM^31,32^ in the resolution of subcellular structures at the submicron level.

### Resolution characterization of VsLFM

Vs-Net can be regarded as a threefold super-resolution network for 4D images (2D spatial domain and 2D angular domain). Although several deep learning-based algorithms have been proposed for single-image super-resolution (SISR) used in fluorescence microscopy^35–37^, they do not consider the phase-correlated angular measurements in LFM. Existing algorithms designed for the 4D spatial–angular domain are still based on the geometric optics used in photography, in which the sampling size is much larger than the diffraction limit^38–41^. Therefore, they are difficult to apply in LFM, which needs to consider the wave-optics diffraction effects for the high-NA objective lens. By contrast, Vs-Net applies multiple designed features in network architecture and adequate light-field datasets in microscopy, which accurately model the large angular disparity and wave-optics diffraction originating from the large collection angle of the objective lens. To show the advantage of VsLFM over state-of-the-art SISR microscopy approaches including the content-aware imaging restoration network (CARE)^35^, deep Fourier channel attention network (DFCAN)^37^, deep Fourier channel attention network with generative adversarial strategy (DFGAN)^37^ and light-field super-resolution approaches including spatial–angular interactive network (LF-InterNet)^40^ and deformable convolution network (LF-DFnet)^41^, we imaged a fixed L929 cell with membrane labeling and compared the high-resolution angular images processed with these networks in terms of signal-to-noise ratio and structural similarity (SSIM) indices (Supplementary Fig. 4). The ground truth data were obtained using sLFM. We then compared the 3D reconstruction results based on the network output. SISR or light-field super-resolution approaches show no additional resolution enhancement after reconstruction, given that the output of different angles does not fulfill the point spread function constraints required for incoherent synthetic aperture (Supplementary Fig. 5). Even when SISR networks were trained on data after reconstruction, the performance was still inferior to that of VsLFM (Supplementary Fig. 6). By contrast, VsLFM has better resolution without artifacts after 3D deconvolution, compared with sLFM.

**Fig. 2:**
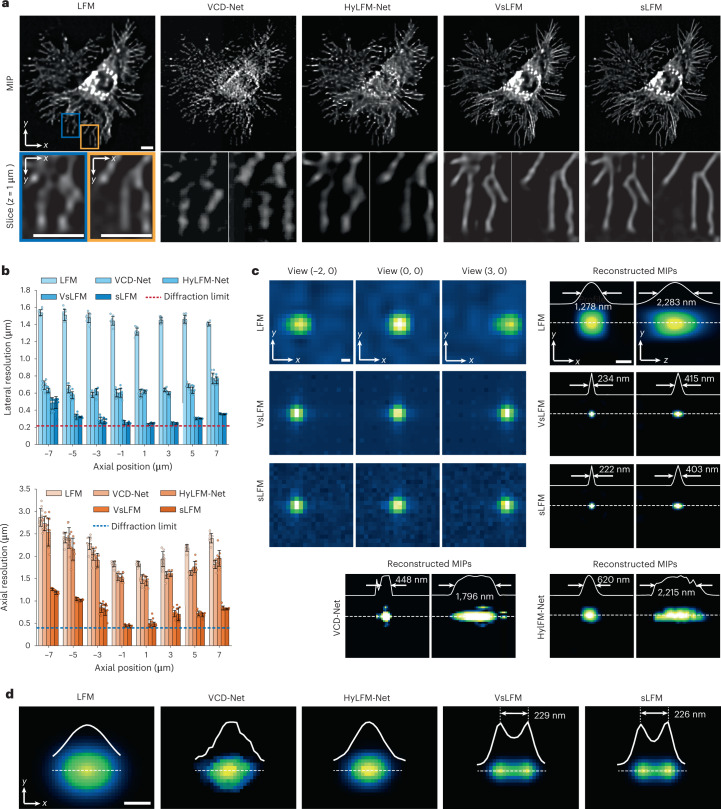
Resolution enhancement of VsLFM. **a**, MIPs and enlarged regions from *xy* slices at *z* = 1 μm of a fixed L929 cell with membrane labeling (TSPAN4-mCherry), obtained by LFM, VCD-Net, HyLFM-Net, VsLFM and sLFM, respectively. **b**, Boxplots of averaged lateral resolution and axial resolution of LFM, VCD-Net, HyLFM-Net, VsLFM and sLFM at different axial positions (*n* = 10 beads per plane). The resolution was estimated by imaging 100-nm-diameter fluorescent beads that were uniformly distributed in low-melt agarose with a ×63/1.4 NA oil-immersion objective, and measuring the FWHM with a Gaussian fit. Lateral and axial diffraction-limited resolutions at a center wavelength of 525 nm are shown with the dashed lines for comparison. Data are presented as mean ± s.d. **c**, Spatial–angular views and the corresponding reconstructed MIPs of a selected 100 nm fluorescent bead, obtained by traditional LFM, VsLFM, sLFM, VCD-Net and HyLFM-Net, respectively. The normalized profiles along the marked dashed lines are shown in the insets. All of the learning-based methods were trained on the bead dataset. **d**, MIPs of two virtually separated beads obtained by LFM, VCD-Net, HyLFM-Net, VsLFM and sLFM with cross-section profiles along the dashed lines. We imaged the same 100 nm bead at two positions with an interval of 230 nm shifted by a piezo translation stage and added the images together to create the two virtually separated beads. Scale bars, 10 μm (**a**), 1 μm (**c**), 200 nm (**d**).

After showing that VsLFM outperforms state-of-the-art super-resolution methods in the spatial–angular domain, we then compared VsLFM with other end-to-end light-field reconstruction networks by evaluating the reconstructed 3D volume (Fig. 2). We chose two recent representative end-to-end networks, VCD-Net^31^ and HyLFM-Net^32^ with optimized parameters (Methods). Much better resolution and contrast could be obtained by VsLFM in terms of the maximum intensity projection (MIP) and single slice (Fig. 2a). We then quantitatively characterized the resolution of VsLFM and other methods by imaging subdiffraction-limit fluorescent beads and measuring the average full width at half-maximum (FWHM) across different axial planes (Fig. 2b). We found that the resolution improvement in VsLFM is at least fourfold higher than that of LFM and twofold higher than that of VCD-Net and HyLFM-Net (Fig. 2c and Supplementary Fig. 7). The stability of VsLFM is also demonstrated by the small variance of FWHM across the whole field of view (FOV) of 210 × 210 × 18 μm^3^. To further verify the resolution improvement of VsLFM, we summed up two images of the same bead by a lateral shift of 230 nm on a piezo stage to generate two virtually separated beads. While LFM with VCD-Net and HyLFM-Net trained on the beads dataset cannot resolve the structures, VsLFM and sLFM can distinguish these two beads clearly (Fig. 2d). In addition, we synthesized 3D distributed tubulins in a numerical simulation to evaluate the resolution of VsLFM for complicated structures (Supplementary Fig. 8a). VsLFM has better performance than LFM, with more high-frequency components in the Fourier spectrum, leading to an improvement in signal-to-noise ratio of ~15 dB and SSIM enhancement of 0.12 in the spatial–angular domain, and an improvement in signal-to-noise ratio of 2 dB and SSIM enhancement of 0.2 after reconstruction (Supplementary Fig. 8b,c). Moreover, the improvement of VsLFM is stable for different sample densities (Supplementary Fig. 8d,e).

To validate the subcellular resolution of VsLFM on biological dynamics in the long term, we imaged mitochondrial (cyan) and membrane (magenta) dynamics in cultured L929 cells with sLFM data as ground truth (Supplementary Fig. 9a and Supplementary Video 1). Remarkable improvements by VsLFM over LFM can be observed via the signal-to-noise ratio and SSIM metrics (Supplementary Fig. 9b–d). Diverse subcellular dynamics can be visualized with low phototoxicity in the long term including mitochondrial behaviors during cell division, fiber retraction, and migrasome formation (Supplementary Fig. 9e,f).

### Robustness in optically challenging environments

Different from in vitro imaging, intravital imaging usually has a large variety of noise and optical aberrations due to the tissue heterogeneity, which makes it challenging for computational microscopy based on accurate imaging models. End-to-end networks are usually limited to specific imaging conditions due to dataset limits, while VsLFM, with its two-step strategy and physics-based priors, can maintain similar performance in complicated scenarios.

**Fig. 3:**
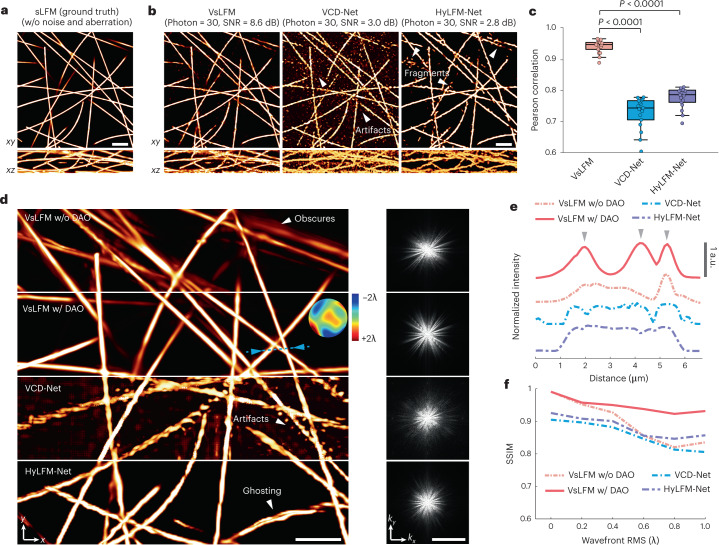
VsLFM shows robustness to noise and optical aberrations. **a**, Orthogonal MIPs of 1-μm-diameter synthetic tubulins, acquired by sLFM with a ×63/1.4 NA oil-immersion objective in ideal imaging conditions, regarded as ground truth. **b**, VsLFM, VCD-Net and HyLFM-Net results after the input contaminated by strong mixed Poisson–Gaussian noise. We set the image bit depth to 16, the variance of Gaussian noise to 5, and the photon number of the maximum intensity to 30. The signal-to-noise ratio (SNR), after reconstruction by different methods, is also given. **c**, Pearson correlations of results obtained by VsLFM, VCD-Net and HyLFM-Net, compared with ground truth. The center line represents the median, the box limits represent the lower and upper quartiles, and the whiskers represent 1.5-fold the interquartile range. *P* values were calculated using the two-sided paired *t*-test: *P* = 9.40 × 10^−18^ for VCD-Net and *P* = 8.77 × 10^−17^ for HyLFM-Net. *n* = 17 for each method, which represents the number of noisy images. **d**, Reconstructed MIPs with an induced aberration wavefront, the root mean square (RMS) of which was set to 1 wavelength, obtained by VsLFM without DAO, VsLFM with DAO, VCD-Net and HyLFM-Net. The estimated wavefront by DAO is shown in the inset. The Fourier transforms corresponding to the whole FOVs by the four methods are shown in the right panel. **e**, Normalized intensity profiles along the blue dashed line marked by the arrows in **d** for four different methods. The arrows indicate the positions of the signal peak. **f**, The curves of reconstructed SSIM versus aberration levels applied for different methods. Note that Vs-Net, VCD-Net and HyLFM-Net used here were all trained on the same tubulin data in ideal imaging conditions without noise and aberration. Scale bars, 10 μm (**a**,**b**), 10 μm (left) and 2 μm^−1^ (right) (**d**).

To evaluate such robustness, we first compared the noise performance of VsLFM, VCD-Net and HyLFM-Net, all of which were trained in high signal-to-noise ratio conditions and tested in photon-limited imaging conditions (Fig. 3a,b and Supplementary Fig. 10). Pre-trained end-to-end networks are very strict on imaging conditions and are prone to overfitting, which confuses the signals with noise, and causes severe artifacts and structural fragments (Fig. 3b). By contrast, Vs-Net learned the physical constraint between angular views, and could effectively distinguish signals from strong noise using the angular–mixed feature to suppress the noise by averaging different angular views (Supplementary Fig. 3). In addition, the iterative tomography using the Richardson–Lucy deconvolution framework, has an inherent denoising capability^42^. As a consequence, VsLFM has better robustness to noise than VCD-Net and HyLFM-Net, and has significantly improved fidelity in low-light conditions (Fig. 3c).

For the aberration problem, we used DAO during the second step of VsLFM. Numerical simulations were conducted to show that VsLFM has similar aberration robustness to sLFM (Supplementary Fig. 11). We trained Vs-Net, VCD-Net and HyLFM-Net on the same synthetic tubulins data in an aberration-free condition, and tested them using different aberration levels (Fig. 3d and Supplementary Fig. 12). Intense aberrations would destroy the mapping relationships learned by end-to-end models, causing visible distortions and artifacts (Fig. 3d), while Vs-Net is robust to the aberrations and DAO can still be applied to the Vs-Net outputs in the second step of iterative tomography for aberration correction. Such a correction is difficult to model in the end-to-end network due to its high-dimensional property. For aberrations with a root mean square of 1 wavelength, VsLFM shows much better resolution and higher SSIM metrics than VCD-Net and HyLFM-Net (Fig. 3e,f). Note that the subsequent in vivo experimental results of VsLFM and sLFM were obtained with DAO, but for simplicity, DAO is no longer specified in the text.

We then imaged a membrane-labeled zebrafish embryo at 3 vps to demonstrate the in vivo subcellular imaging capability of VsLFM (Supplementary Fig. 13a–f and Supplementary Video 2). The spatial heterogeneity in multicellular organisms and the sensitivity to photodamage lead to severe shot noise and optical aberrations. Enlarged views show the elaborate dynamics of fiber movements over 20 minutes, with narrower intensity profiles of VsLFM results than those of LFM (Supplementary Fig. 13g–i). These results further corroborate the stable resolution and contrast improvement by VsLFM in complicated environments.

### Generalization over diverse structures and magnifications

The generalization ability is one of the most critical problems in the biological applications of deep learning, especially in cross-sample experiments with a large data diversity. Moreover, it is very difficult to collect a huge dataset to cover diverse biological phenomena, for example, even for a specific type of cell during different physiopathological states. VCD-Net and HyLFM-Net, which rely heavily on the data prior, work well on a similar type of data but cannot make accurate predictions on unseen data. Vs-Net was designed to learn the physical prior between phase-correlated angular components rather than texture data priors only, leading to a better generalization ability for different sample structures than previous end-to-end networks. To verify such a capability, we compared VsLFM with VCD-Net and HyLFM-Net using different datasets for training during simulation. All of them showed good reconstruction performance when the test datasets and the training datasets were both based on synthetic tubulins (Supplementary Fig. 14a,b). However, if these network models were trained on bead data and tested on tubulins data, the performance of VCD-Net and HyLFM-Net dropped dramatically in terms of the Pearson correlations compared with ground truth. Structural artifacts with similar shapes to the training data could be observed in the results of the end-to-end networks (Supplementary Fig. 14c). By contrast, VsLFM exhibited stable performance without reconstruction artifacts (Supplementary Fig. 14c–e).

**Fig. 4:**
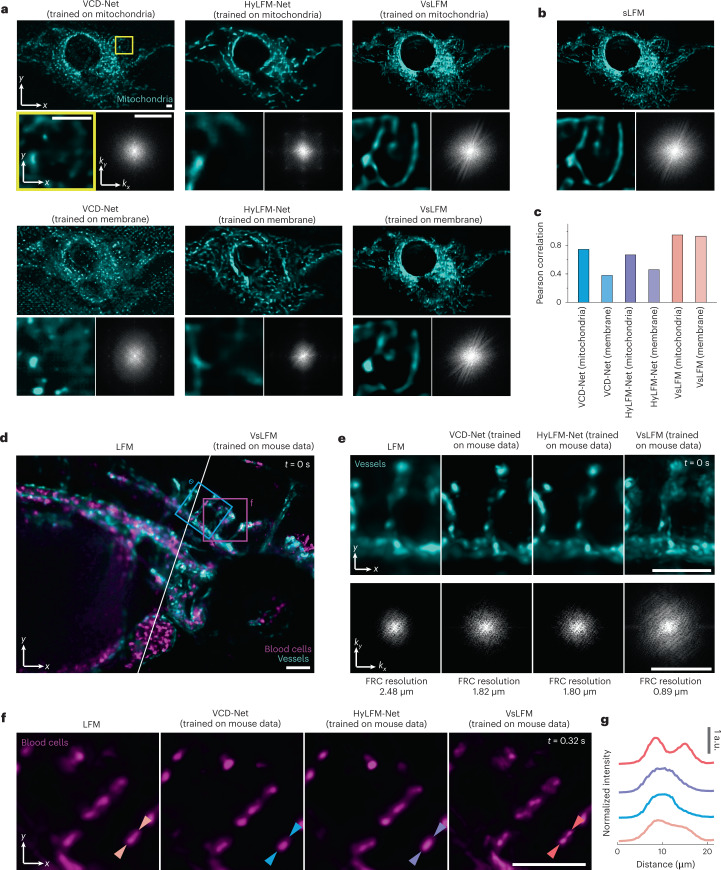
VsLFM has better generalization ability than end-to-end networks. **a**, MIPs and enlarged views of a fixed L929 cell with mitochondria labeling (TOM20-GFP), obtained by VCD-Net, HyLFM-Net and VsLFM trained on the same type of sample (mitochondria, upper row) and a different type of sample (membrane, lower row). The corresponding Fourier spectrum is shown in the bottom-right corner of each panel. **b**, Corresponding sLFM results as ground truth. **c**, Bar chart of Pearson correlations between the results of the mitochondria channel obtained by sLFM and the results obtained by VCD-Net, HyLFM-Net and VsLFM trained on different datasets. **d**, MIPs acquired by LFM (left) and VsLFM (right) of circulating blood cells (magenta) and vessels (cyan) in a zebrafish larva. The data were captured with a ×20/0.5 NA air objective at 50 vps. Given that the ground truth data cannot be obtained in this highly dynamic sample, the network models were trained on mouse liver data with vessel and neutrophil labeling and captured by a ×63/1.4 NA oil-immersion objective. **e**, Enlarged MIPs of the vessel channel marked by the blue box in **d** at *t* = 0 s, acquired by different methods. The corresponding Fourier transforms of the MIPs with estimated resolutions by Fourier ring correlation (FRC) are shown in the bottom row to indicate the resolution enhancement by VsLFM. **f**, Enlarged MIPs of the blood cell channel marked by the magenta box in **d** at *t* = 0.32 s. **g**, Normalized intensity profiles along the lines indicated by the arrows in **f**, showing that the two adjacent blood cells that could not be distinguished by previous methods were resolved by VsLFM. Scale bars, 5 μm (top, bottom left, **a**) and 3 μm^−1^ (bottom right, **a**), 50 μm (**d**,**f**), 50 μm (top) and 1 μm^−1^ (bottom) (**e**).

We then compared the generalization ability of VsLFM with that of VCD-Net and HyLFM-Net in a cross-channel experiment. We made predictions on the mitochondria channel of an L929 cell using two network models pre-trained on the mitochondria channel and the membrane channel, respectively (Fig. 4a). The zoom-in regions and corresponding Fourier spectra show that VsLFM has stable performance for different sample structures, while VCD-Net and HyLFM-Net show resolution degradation and artifacts during the generalization process. The results obtained by sLFM were used as the ground truth to calculate the Pearson correlation (Fig. 4b). The reconstruction fidelity decreased quickly during generalization for previous end-to-end networks, but remained stable for VsLFM (Fig. 4c).

The generalization capability is critical for highly dynamic samples, given that we cannot capture ground truth data for training. A typical example is flowing cells in the blood circulation system. To show the advantage of VsLFM, we imaged a zebrafish larva labeled with blood cells and vessels, which was anesthetized and embedded in agarose gel during imaging with a ×20/0.5 NA air-immersion objective at 50 dual-color vps (Fig. 4d and Supplementary Video 3). We trained the Vs-Net on mouse liver data with vessel and neutrophil labeling captured by another objective with a ×63 magnification and an NA of 1.4 to predict the high-resolution structures of vessel membranes and blood cells in zebrafish. Although the network was trained on different species and different imaging magnifications, VsLFM exhibited stable performance with better resolution^43^ than the other methods (Fig. 4e). Two adjacent flowing cells that are indistinguishable in one frame can be clearly recognized using VsLFM even with a high motion speed (Fig. 4f,g). Owing to the enhancement in both spatial resolution and temporal resolution by VsLFM, 76 flowing blood cells with reduced crosstalk can be easily tracked in three dimensions in the extremely short imaging duration of 0.40 s (Supplementary Fig. 15). Therefore, VsLFM can serve as a promising tool for high-fidelity downstream studies of the circulatory system and hemodynamics in diverse model organisms^44^.

### Robust high-resolution snapshot 3D imaging in mammals

**Fig. 5:**
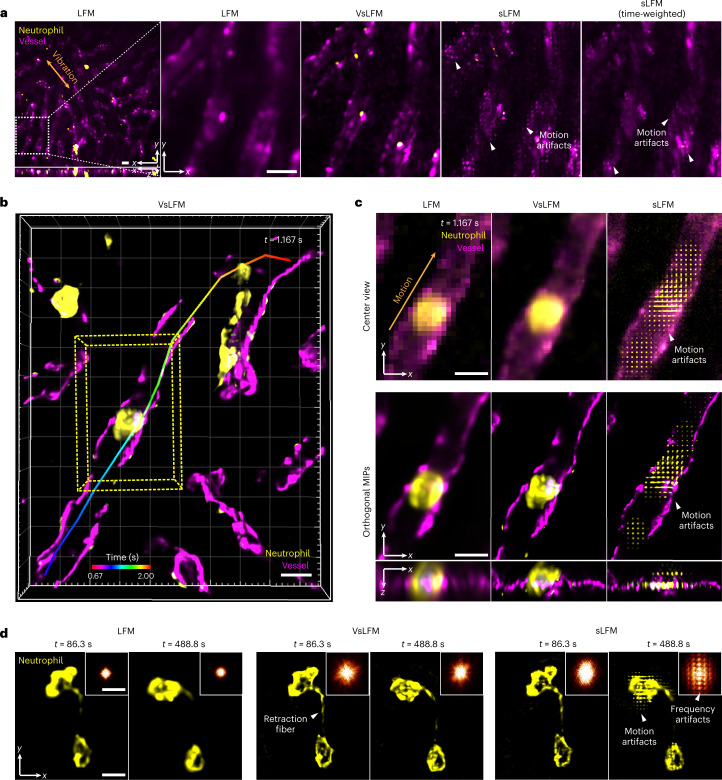
Long-term high-speed imaging of subcellular dynamics in living mouse livers. **a**, Whole-FOV and enlarged MIPs of neutrophils and vessels in a living mouse liver with strong motions induced by respiration, obtained by LFM, VsLFM, sLFM and sLFM with the time-weighted algorithm, respectively. **b**, MIP of a neutrophil washed away by the blood flow in vessels, which was captured by VsLFM at 12 vps. The tracked trace obtained with Imaris 9.0.1 software was overlaid with the temporal information coded in different colors. The overall flow duration is 1.33 s, from *t* = 0.67 s to *t* = 2.00 s. **c**, Center view of spatial–angular components and corresponding reconstructed MIPs of marked regions in **b** at *t* = 1.167 s, obtained by LFM, VsLFM and sLFM, respectively. The orange arrow indicates the movement direction and the white arrows indicate the motion artifacts in sLFM. **d**, MIPs of neutrophils with high-speed migration in a living mouse liver, obtained by LFM, VsLFM and sLFM, respectively. The neutrophil at *t* = 86.3 s moved slowly without motion artifacts, and the retraction fiber could be observed with high resolution by both VsLFM and sLFM. However, at *t* = 488.8 s the neutrophil migrated at high speed, leading to visible motion artifacts in the sLFM results. The upper-right insets show the corresponding Fourier transform of the MIPs, which also show the periodic frequency patterns caused by sample motion in the sLFM results. Meanwhile, VsLFM effectively eliminates motion artifacts with high spatial resolution. Scale bars, 10 μm (**a**–**c**), 10 μm (main) and 3 μm^−1^ (inset) (**d**).

Given that VsLFM facilitates snapshot near-diffraction-limited 3D imaging with broad generalization, it can analyze subcellular dynamics in complicated dynamic mammalian environments involving the beating heart, respiration and blood flow, especially for organs close to the heart or lung. Such strong motions in some frames would perturb the physical scanning pattern of sLFM during the 9-image acquisition and cause motion artifacts and reduction of temporal resolution in sLFM for highly dynamic samples. To demonstrate this advantage of VsLFM, we imaged endogenous neutrophils and vessels in living mouse livers during their native physiological processes. The mice were anesthetized and dissected to expose the liver on the coverslip. To reduce respiration-induced motions in sLFM, a region close to the tip of the liver needs to be used, which in turn limits the effective imaging area. Although a time-weighted algorithm has been developed for sLFM to compensate for the loss of temporal resolution, movements that are too fast would still exceed the adjustable range of the algorithm and degrade the imaging performance^20,45^ (Fig. 5a). The LFM results exhibited no artifacts at the expense of low spatial resolution, while VsLFM improved the resolution without motion artifacts (Fig. 5a and part I of Supplementary Video 4). To verify the fidelity, we compared the results of VsLFM and sLFM on the same frame without motion and noted a similar subcellular resolution (Supplementary Fig. 16). The whole process of a neutrophil gradually generating a retraction fiber, accompanied by periodic 3D vibrations of the whole FOV, was clearly observed at subcellular resolution by VsLFM, demonstrating its robust performance in a complicated environment (Supplementary Fig. 16 and Supplementary Video 5).

Even in a stable environment, cellular dynamics in blood vessels still involve a large variety of velocities during different states. We captured another neutrophil that was washed away by the blood flow in a vessel at 12 vps, which was extremely fast and lasted for only approximately 1.33 s across approximately 100 μm in three dimensions (Fig. 5b and part II of Supplementary Video 4). VsLFM outperformed LFM with better resolution and contrast, and concurrently eliminated severe motion artifacts in sLFM (Fig. 5c). In addition, VsLFM can simultaneously retain intact cell shape and maintain subcellular resolution during fast neutrophil migration, enabling fine structures such as retraction fibers to be resolved distinctly without being influenced by the motion artifacts (Fig. 5d and part III of Supplementary Video 4). By using a single LFM image for high-resolution structures, the phototoxicity of VsLFM can be further reduced by ninefold for even longer imaging durations than sLFM.

### Ultrafast high-resolution 3D voltage imaging in *Drosophila*

Intravital imaging of voltage activities are important for the study of learning units with feedback interconnections and complex interactions between short-term and long-term memory in *Drosophila* brains^46^. However, it has long been a challenge to capture the voltage dynamics in vivo at subcellular resolution across a large volume due to the extremely fast transients, which occur usually over 200 Hz, and the low signal-to-noise ratio of the voltage indicators with short exposure time. VsLFM could address this problem with its capability of snapshot high-resolution 3D imaging and low phototoxicity.

**Fig. 6:**
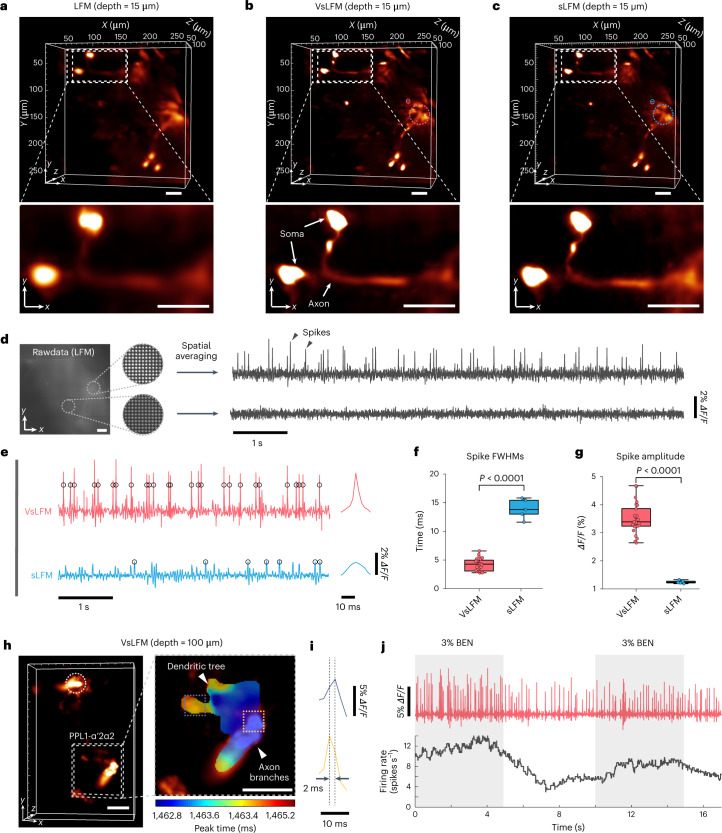
In vivo high-resolution volumetric voltage imaging of sparsely labeled neurons in *Drosophila* at 500 vps. **a**–**c**, 3D rendering volumes and enlarged MIPs of PPL1 dopamine neurons at a depth of 15 μm in *Drosophila* brain (*MB065B-GAL4* > *20×UAS-pAce*), obtained by LFM (**a**), VsLFM (**b**) and sLFM (**c**). **d**, Average temporal traces extracted from two different regions in the raw light-field images. **e**, Left, temporal traces extracted from the manually selected region in **b** and **c** for VsLFM and sLFM, with the black circles marking the identified spikes. Right, corresponding average waveforms for the spikes. The data of sLFM and VsLFM were collected sequentially on the same *Drosophila*, therefore the spontaneous voltage activities occurred at different time stamps. **f**,**g**, Comparisons of the temporal FWHMs (**f**) and amplitudes (**g**) of the spikes between VsLFM and sLFM in the same selected region as **e**. The center line represents the median, the box limits represent the lower and upper quartiles, and the whiskers represent 1.5-fold the interquartile range. *P* values were calculated using the two-sided paired *t*-test. *P* = 1.77 × 10^−15^ (**f**) and *P* = 8.14 × 10^−11^ (**g**). *n* = 5 for the sLFM results and *n* = 26 for the VsLFM results, where *n* represents the number of identified spikes. **h**, 3D rendering volume of PPL1 dopamine neurons at a depth of 100 μm in another *Drosophila* (*MB065B-GAL4*>*20×UAS-pAce*) obtained by VsLFM, with the enlarged time-coded MIPs. Different colors represent the peak instants of the voltage signal for every pixel during 2.4 ms. **i**, Voltage spikes extracted from two regions in the PPL1-α‘2α2 neuron, showing a 2 ms delay in action potentials. **j**, Upper row, odor-evoked voltage traces extracted from the region marked by the white dashed circle in **h**. Bottom row, corresponding time-dependent firing rates. The gray rectangles indicate the time window when we applied the 3% benzaldehyde (BEN) stimulus. Scale bars, 30 μm (**a**–**d**, **h**).

To demonstrate its advantages over traditional LFM and sLFM, we constructed an upright sLFM system with a high-speed scientific camera to observe the 3D voltage transients of sparsely labeled dopamine neurons across the whole brain of awake behaving *Drosophila* (*MB065B-GAL4*>*20×UAS-pAce*)^46,47^ at 500 vps. We imaged the same sample by LFM and sLFM sequentially for comparison. The VsLFM results were obtained from the LFM data. Both VsLFM and sLFM have comparable resolution and clearly resolve neural axons at a depth of 15 μm with more elaborate detail than LFM (Fig. 6a–c). In addition, many action potentials can be visualized by averaging a large local region in the raw LFM measurements while the temporal trace of another region shows no apparent fluctuations, indicating the fidelity of the voltage signals (Fig. 6d). However, sLFM lacked sufficient temporal resolution to detect the action potentials due to the 9-image sliding window for physical scanning, which stretched the spike widths and reduced the response amplitudes (Fig. 6e). Some low-amplitude spikes may also be neglected. By contrast, VsLFM harnessed the advantages of both LFM and sLFM. With the snapshot property, VsLFM maintained high temporal resolution to resolve the action potentials at subcellular resolution across a large volume of ~260 × 260 × 100 μm^3^ at 500 vps, enabling concurrent neural recording of multiple brain regions in the *Drosophila* (Fig. 6e and part I of Supplementary Video 6). Quantitative analysis showed that VsLFM distinguished the voltage spikes accurately with a significantly smaller temporal FWHM of approximately 5 ms (Fig. 6f) and obtained an at least twofold improvement in spike amplitude relative to sLFM (Fig. 6g).

We then used VsLFM to record the 3D voltage activities in PPL1 dopamine neurons at 500 vps in *Drosophila* using 3% benzaldehyde as a repulsive odor stimulus. The observed region was located around 100 μm below the optical window in the cuticle of an adult *Drosophila* (*MB065B-GAL4*>*20×UAS-pAce*). With the ultrahigh spatiotemporal resolution of VsLFM, the 3D propagation of action potentials in axon branches and dendritic tree of the PPL1-α‘2α2 neuron can be detected (Fig. 6h,i and part II of Supplementary Video 6). We also observed an increase in firing rates during the stimulus, which accords well with a previous study^46^ (Fig. 6j). With the ultrahigh 3D imaging speed and low phototoxicity, VsLFM facilitates broad study of neural activity with the help of advanced voltage indicators, which would be difficult for previous imaging methods.

## Discussion

Here, we have developed a physics-based virtual-scanning framework for LFM to enhance the spatial resolution by fourfold with broad generalization, enabling snapshot intravital volumetric imaging with subcellular resolution and a low phototoxicity of only several mW mm^−2^ at a camera frame rate up to 500 vps. VsLFM and sLFM are not conflicting but are complementary. The main problem with sLFM is the motion artifacts and the reduction of temporal resolution due to physical scanning in highly dynamic conditions such as the beating heart, blood flow and neural activities. By using the ground truth data captured by sLFM without motion artifacts for the training of VsLFM, VsLFM provides the ultrafast high-resolution 3D imaging for highly dynamic samples or LFM without the scanning module.

Physics-based VsLFM addresses three major problems in previous end-to-end networks in LFM, including the huge resolution gap between the raw LF measurements and the diffraction limit of the objective, sensitivity in optically challenging conditions, and low generalization ability across diverse structures, species and imaging systems. Using the physical priors of frequency aliasing and point spread function models rather than texture priors only, VsLFM enhances the spatial resolution up to the diffraction limit and improves the generalization ability for a wide range of applications (Figs. 3, 4 and Supplementary Fig. 14). The output of Vs-Net is robust to optical aberrations and compatible with the DAO technique to correct optical aberrations during 3D reconstruction, which is difficult to model in the end-to-end networks. By focusing on the upsampling process to address the frequency aliasing problem, VsLFM is also compatible with other 3D reconstruction algorithms or neural networks. To reduce the computational costs of deconvolution, we developed a new end-to-end reconstruction network named HyLFM-A-Net, extended from the existing HyLFM-Net^32^ for the output of Vs-Net. We introduced a channel attention mechanism^48^ into the existing HyLFM-Net to replace iterative tomography for fast reconstruction (Supplementary Fig. 17 and Methods). However, the robustness to sample aberration will decrease for complicated imaging environments given that all of these end-to-end networks do not consider the influence of optical aberrations. Better neural networks for reconstruction, such as self-supervised methods, can be designed in the future with more physical priors. Moreover, given that the confocal or light-sheet set-ups can physically address the missing-cone problem, training the 3D reconstruction network for VsLFM with paired confocal or light-sheet data can increase the optical sectioning at the cost of data generalization. Structured illumination may be another choice to improve the optical sectioning and depth penetration for VsLFM^49^.

VsLFM could also work for LFM equipped with different microlens arrays through parameter adjustment and retraining with a detailed guide (Methods and Fig. 6). When the microlens array parameters do not change much, the Vs-Net can be used with simple preprocessing of angular interpolation (Supplementary Fig. 18). Therefore, the virtual-scanning framework with our pre-trained model could be applied to the data captured by different types of unfocused LFM in previous studies. Transfer learning can be applied to further increase the performance on specific data and accomplish a faster convergence with only a small amount of additional data required (Supplementary Fig. 19). In the meantime, better neural networks can be designed in the future based on our open-source 40 GB sLFM dataset of more than 1,300 pairs of low-resolution and high-resolution light-field images from multiple species, structures and imaging conditions. This VsLFM adopts the unfocused form to obtain a large axial range with extended depth of field, but it is still limited by the objective numerical aperture^50^. The use of objective lenses with a lower numerical aperture would improve axial range at the cost of spatial resolution. Improvement of the angular resolution^20^ or phase modulation at the pupil plane^51^ is also anticipated to increase the axial coverage. As a one-photon fluorescence microscopy technique, the imaging penetration capability of VsLFM is fundamentally limited by tissue scattering and background fluorescence, which can be enhanced with other scattering removal methods^27^. Nevertheless, with superior resolution enhancement and broad generalization, this physics-based virtual-scanning mechanism in LFM fulfills the requirement for extremely high-speed intravital imaging of subcellular structures across a large FOV with minimized phototoxicity, further broadening the versatility and practical applications of LFM in challenging complicated environments.

## Methods

### VsLFM set-up and data collection

The inverted sLFM optical system was built in accordance with our previous research^20^. A standard inverted fluorescence microscope (Zeiss, Observer Z1) was used as a basic imaging module, configured with a ×63/1.4 NA oil-immersion objective (Zeiss Plan-Apochromat ×63/1.4 NA Oil M27) and a ×20/0.5 NA air-immersion objective (Zeiss EC Plan-Neofluar ×20/0.5 NA M27) for different experiment requirements. A microlens array with the pitch size of 100 μm and focal length of 2,100 μm modulated the emission light into a 4D light field, with a 2D galvo system to shift the image plane periodically at high speed. Each microlens covered 13 × 13 sensor pixels for angular sampling. Multi-channel lasers (Coherent OBIS 405/488/561/640) and a scientific camera (Andor Zyla 4.2 Plus PCIE) were used for fluorescence excitation and data collection. All hardware synchronization of the system was carried out using a National Instruments (NI) control box (NI USB-6363) and LabVIEW software (2019 version), which were integrated in an acquisition graphical user interface named sLFdriver, as described in the published protocol^45^. To construct the training dataset, scanning light-field images with the scanning period of 3 × 3 (2,048 × 2,048 pixels each) were captured by sLFM. Then, the light-field image taken in the middle of the series was extracted as the paired low-resolution light-field image. Next, a pixel-realignment algorithm was performed on the single light-field image and scanning light-field images to yield the paired low-resolution and high-resolution spatial–angular views, which were regarded as the input and target for network training. For example, in the inverted sLFM system, the low-resolution spatial–angular views consist of 153 × 153 spatial pixels and 13 × 13 angular pixels, and the high-resolution spatial–angular views consist of 459 × 459 spatial pixels and 13 × 13 angular pixels, which are determined by the parameters of the microlens array and camera. We also constructed an upright sLFM system for imaging voltage activities in *Drosophila* brains, configured with a ×25/1.05 NA water-immersion objective (Olympus XLPLN25XWMP2) and a high-speed scientific camera (Teledyne Photometrics Kinetix). In the implementation of the upright system, a customized microlens array with a pitch size of 136.5 μm and a focal length of 2,800 μm was used to cover 21 × 21 angular pixels for a large axial coverage. During the *Drosophila* experiment, the camera pixel region was set to 2,000 × 2,000, with LFM data containing 91 × 91 spatial pixels and 21 × 21 angular pixels, and sLFM data containing 273 × 273 spatial pixels and 21 × 21 angular pixels. When capturing test data for VsLFM, the 2D galvo was set to its offset voltage and kept stable as in traditional LFM. Then the system captured single-frame or time-lapse light-field data, and further realigned them into low-resolution spatial–angular views, of which spatial resolution would be improved by the virtual-scanning framework. Each frame of VsLFM was collected within a snapshot. For intuitive comparisons between VsLFM and sLFM, the unscanned light-field images were directly extracted from scanning light-field images. The snapshot images were used to derive the results of VsLFM, and the sLFM results were used as the paired ground truth for performance comparison. The data used in testing were not involved in network training. Detailed imaging conditions for all of the fluorescence experiments in this study, including the fluorescent label, exposure time, excitation power, volume rate and objective, are listed in Supplementary Table 2.

### Virtual-scanning network

In our proposed Vs-Net, the input is a 3D tensor of low-resolution spatial–angular views (rearranged into the form of height × width × angle), while the output is a 3D tensor of high-resolution spatial–angular views. For the preprocessing of data captured by the inverted system, the training dataset was partitioned into small patches with the input size of 25 × 25 × 169 pixels, and the output and target size of 75 × 75 × 169 pixels. The input and corresponding target data were normalized by the average value of maximum intensities from different time-lapse data. An overview of Vs-Net architecture is given in Supplementary Fig. 2a and the detailed network parameters for each layer are listed in Supplementary Table 1. In our implementation, Vs-Net emphasizes the spatial–angular feature and angular–mixed feature on the basis of a global residual network containing feature extraction, interaction and fusion modules^40^. The input spatial–angular views (size of 25 × 25 × 169 pixels, height × width × angle) are first fed into the feature extractor to generate spatial–angular features (size of 169 × 25 × 25 × C pixels, angle × height × width × channel), light-field features (size of 325 × 325 × C pixels, height × width × channel) and angular–mixed features (size of 25 × 25 × C pixels, height × width × channel), where C denotes the channel number of 2D convolution layers and is usually set to 64. The spatial–angular feature is extracted to better consider the phase correlation induced by the diffraction effect of the small microlens aperture and the disparity between different angular views in LFM, which is much larger than that used in macroscale light-field photography due to the large collection angle of the objective lens. The light-field feature is generated for comprehensive consideration of the angular information at different local spatial regions. The angular–mixed feature, weighted by multiple angular views, is used to reinforce the fidelity of fine structures under strong noise conditions. We use only linear operations to reshape and decouple the input into features of these three domains, leaving non-linear activation and deeper layers to the subsequent interaction and fusion stage to obtain more expressive features. Note that the linear operations such as pixel alignment and dilated convolution should have the appropriate parameters associated with the LFM configuration and data structure. The three features are then passed through the feature interaction and fusion modules to enable multiple information interaction and integration. The detailed structures of the interaction and fusion modules are shown in Supplementary Fig. 2b. The light-field interaction module plays the major role in enhancing the spatial resolution, while the spatial–angular feature and the angular–mixed feature are considered as complementary information and interact with the light-field feature for spatial super-resolution, to provide sufficient consideration of phase correlation between different angles and to reinforce the fidelity of fine structures under strong noises. During the learning process, the light-field feature is interactively fused with the spatial–angular feature and the angular–mixed feature in the spatial–angular interaction module and the angular–mixed interaction module, respectively. We apply a local residual connection in the output features of the aforementioned interaction modules to fully extract the features. The ablation study demonstrates the effective collaboration of the interaction modules and verifies the functions of the three features (Supplementary Fig. 3). The feature interaction modules are followed by a K cascade and concatenation module, where K denotes the cascaded number, usually set to 4. Next, the concatenated interacted spatial–angular feature (size of 169 × 25 × 25 × K·C pixels, angle × height × width × K-fold channels) and angular–mixed feature (size of 25 × 25 × K·C pixels), are realigned into the light-field domain and subsequently squeezed into C channels before concatenating with K light-field features (size of 325 × 325 × K·C pixels), to yield the fully concatenated interacted features (size of 325 × 325 × (K + 2)·C pixels). The concatenated interacted features are fused by a 1 × 1 convolution layer and a leaky rectified linear unit (Leaky ReLU) layer to generate the fused light-field feature (size of 325 × 325 × C pixels). Last, the fused features are fed into the upsampling module with a pixel shuffle to be scaled up by 3 to produce the high-resolution spatial–angular views (size of 75 × 75 × 169 pixels, height × width × angle). In addition, a global residual connection is used by adding the output and the upsampled input with bicubic interpolation, to fully recover the high-frequency details and speed up the convergence.

For network training we typically used 5,000 paired spatial–angular patches of the same dataset, and it usually took approximately 40 epochs for network convergence. Considering the inherent sparsity of fluorescent specimens^52^, the pixel-wise mean absolute error (L1-norm error) is adopted as the loss function, which could be expressed as:

$${\mathrm{loss}} = \left\| {X - Y} \right\|_1,$$

where *X* denotes the ground truth of spatial–angular views and *Y* denotes the output spatial–angular views. The parameters of the Adam optimizer were set to *β*_1_ = 0.9, *β*_2_ = 0.999. The learning rate was initialized to 2 × 10^−4^ and then decreased by a factor of 0.5 for every 10 epochs during the training process.

Vs-Net works well for different types of data and has the robust flexibility of input size. After the network is trained, it can be applied to specimens from different organism. For the inference process, the input data with the size of 153 × 153 × 169 pixels are first partitioned into nine partially overlapping patches with the size of 69 × 69 × 169 pixels, and then transformed to nine high-resolution outputs with the size of 207 × 207 × 169 pixels, using sigmoid-based image fusion^53^ to generate the final output with the size of 459 × 459 × 169 pixels. Finally, the output spatial–angular views are used to obtain the high-resolution volume by iterative tomography with DAO, which has been described in the previous work^20^. The validity of Vs-Net has been verified in extensive fluorescence specimens including fluorescent beads, living cells with mitochondria and membrane labeling, blood cells in zebrafish larvae, immune cells in mouse livers, and voltage indicators in *Drosophila*. To validate the considerable scalability and generalization capability of Vs-Net, both numerical simulations and biological experiments were performed. First, we performed a generalization test on cross-channel tasks, in which the mitochondria channel and membrane channel in L929 cells were mutually trained and predicted (Fig. 4a–c). Second, a Vs-Net model that was pre-trained on mouse liver data with vessel and neutrophil labeling under a ×63/1.4 NA oil-immersion objective, performed well on highly dynamic membrane and blood cells in a living zebrafish larva, which were captured by another air-immersion objective (Fig. 4d–g and Supplementary Fig. 15). Third, synthetic specimens of beads and tubulins, which have great morphological differences, were selected for cross-sample and transfer learning experiments (Supplementary Figs. 14 and 19).

The Vs-Net can work on data captured by LFM with different kinds of microlens arrays through slight network modifications. To enable Vs-Net to accommodate data with different angular pixels, we need to modify the parameter of angular numbers of the network, while the main architecture of the network remains the same as before. To verify it, we set up an upright system, in which another microlense array with the pitch size of 136.5 μm and focal length of 2,800 μm was used. The data obtained by the upright system have 21 × 21 angular views, whereby the input size of the training dataset patches is 25 × 25 × 441 pixels (height × width × angle) and the output and target sizes are 75 × 75 × 441 pixels. Correspondingly, the spatial–angular feature has the size of 441 × 25 × 25 × C pixels (angle × height × width × channel), the light-field feature has the size of 525 × 525 × C pixels (height × width × channel) and the angular–mixed feature has the size of 25 × 25 × C pixels (height × width × channel), where C is usually set to 64 or 32, dependent on the GPU (graphics processing unit) memory. After network training, the test data can be enhanced by Vs-Net. The *Drosophila* data in Fig. 6 were processed by the Vs-Net with 21 × 21 angular views as input. The VsLFM results have comparable resolution to those of sLFM when there were no motion artifacts.

The network was implemented on a PyTorch platform with two NVIDIA RTX 2080 Ti GPUs. The whole training process for 40 epochs on a typical training set (approximately 5,000 pairs) took approximately 16 h, and inference and post-processing on one whole-FOV light-field image took approximately 5 s for a spatial–angular image size of 459 × 459 × 169 pixels. Training and inference time can be further reduced by using more powerful GPUs. To maximize its accessibility, we have released Vs-Net codes and corresponding 3D reconstruction scripts with demonstration data to promote interdisciplinary research.

### Comparison with previous methods

We compared our method with the previous methods, such as traditional LFM, sLFM, CARE, DFCAN and DFGAN, LF-InterNet, LF-DFnet, VCD-Net and HyLFM-Net. All traditional LFM results used in this work were reconstructed using phase-space deconvolution with a simple bicubic interpolation applied on the low-resolution spatial–angular views^25^. All sLFM results were acquired as described in the original study^20^, and all in vivo biological results were obtained with DAO, but for simplicity, DAO is no longer specified in the texts.

For comparison with CARE^35^, DFCAN^37^ and DFGAN^37^, we adopted two training strategies for comprehensive evaluations. First, we trained the networks on the same spatial–angular views used in Vs-Net, which were split into two stacks of images consisting of low-resolution and high-resolution pairs (Supplementary Figs. 4 and 5). For CARE, we used the bicubic interpolation to upsample the low-resolution images by a factor of 3 and cropped them into patches with 128 × 128 pixels (height × width) as input, and the corresponding high-resolution images were also cropped into patches with 128 × 128 pixels as targets. For the training of DFCAN and DFGAN, the low-resolution data were cropped into patches with 64 × 64 pixels as input, and high-resolution resolution data were cropped into patches with 192 × 192 pixels as targets. The scale factor was adjusted to 3. The training process took approximately 10 h for CARE, 12 h for DFCAN and 18 h for DFGAN on a single NVIDIA RTX 2080 Ti GPU for convergence. After network inference, the output images were re-stacked as spatial–angular views according to their angular positions, and the final whole-FOV results were obtained with the same sigmoid-based image fusion used in Vs-Net. The results of the comparisons are shown in Supplementary Figs. 4 and 5. Second, we also trained the networks based on the reconstructed volumes of LFM and sLFM for comparison (Supplementary Fig. 6). In this case, the data pairs consisted of the low-resolution volumes of LFM (with the size of 1,989 × 1,989 × 101 pixels, height × width × depth) and the high-resolution volumes of sLFM (with the size of 1,989 × 1,989 × 101 pixels). For CARE, the data pairs were cropped into 3D patches with the size of 128 × 128 × 16 pixels for training. For DFCAN and DFGAN, which are designed for SISR tasks, the 3D volume pairs were segmented as a stack of images. The input low-resolution data were downsampled by a factor of 3 and cropped into patches with the size of 128 × 128 pixels, while high-resolution targets were cropped into patches with the size of 384 × 384 pixels. The training processes of CARE, DFCAN and DFGAN were performed on a single NVIDIA RTX 2080 Ti GPU, which took approximately 20 h for CARE, 25 h for DFCAN and 40 h for DFGAN for convergence. After network inference the outputs were stitched using sigmoid-based image fusion working in 3D or 2D space.

LF-InterNet^40^ requires raw light-field images as input and high-resolution spatial–angular images as targets, while LF-DFnet^41^ requires tiled low-resolution spatial–angular images as input and tiled high-resolution spatial–angular images as targets. The training inputs used for Vs-Net with the size of 25 × 25 × 169 pixels (height × width × angle) were transformed to light-field images with the size of 325 × 325 pixels (height × width) for LF-InterNet as input, and tiled to images with the size of 325 × 325 pixels (height × width) for LF-DFnet as input. Correspondingly, the high-resolution spatial–angular images from the Vs-Net dataset were tiled to images with the size of 975 × 975 pixels (height × width) as targets for both LF-InterNet and LF-DFnet. The tiling operation stitches images from different angles into a 2D image according to their angular positions in the way of a montage. The whole training process of LF-InterNet and LF-DFnet took approximately 14 h and 20 h, respectively, on a single NVIDIA RTX 2080 Ti GPU for convergence. After network inference the outputs were rearranged according to the spatial–angular domain, and stitched by the same sigmoid-based image fusion used in Vs-Net. The results are compared in Supplementary Figs. 4 and 5.

VCD-Net^31^ and HyLFM-Net^32^ require light-field images as input data and corresponding confocal or light-sheet volumes as targets, to train fully supervised models. However, the target volumes are difficult to acquire if using a simple LFM or sLFM system. To compare VsLFM with VCD-Net and HyLFM-Net, we first conducted numerical simulations in which the required confocal or light-sheet volumes could be replaced by the original synthetic volumes, to quantitatively evaluate their performance in complicated environments (Fig. 3 and Supplementary Fig. 14). To mimic the practical situations for fair comparison between different methods, we trained Vs-Net, VCD-Net and HyLFM-Net in the same ideal imaging conditions, and performed network inferences in multiple complicated scenes. We also compared VsLFM with VCD-Net and HyLFM-Net on experimental data (Figs. 2, 4 and Supplementary Fig. 7). Given that the confocal and light-sheet modules are difficult to integrate into the sLFM system, the high-resolution volumes acquired by sLFM with 3D reconstruction were used as training labels. The VCD-Net and HyLFM-Net results in Figs. 2–4 and Supplementary Figs. 7, 14 were obtained using open-source codes in previous studies^31,32^, and the corresponding parameter of angle number was adjusted to make it suitable for our system implementations. Specifically, the number of input channels of VCD-Net and HyLFM-Net was modified to 169, and a bicubic interpolation layer was attached to the end of each network to match the output volume size of the target. The input data are the same as that used in Vs-Net, with the size of 153 × 153 × 169 pixels. For network training we randomly cropped out small input patches with the size of 40 × 40 × 169 pixels (for HyLFM-Net) and 64 × 64 × 169 pixels (for VCD-Net), as well as the corresponding volume regions, to create data pairs. The networks required to be trained for around 200 epochs for convergence. For network inference, partially overlapped patches with the size of 80 × 80 × 169 pixels (for HyLFM-Net) and 64 × 64 × 169 pixels (for VCD-Net) were cropped from input data. The same sigmoid-based image fusion used in Vs-Net was adopted to stitch the output sub-volumes into whole-FOV volumes. The whole training process of VCD-Net and HyLFM-Net took approximately 32 h, and the inference time for one whole-FOV frame took approximately 3 s for VCD-Net and 7 s for HyLFM-Net. The network training and inference were done on a single NVIDIA RTX 2080 Ti GPU.

We also develop a new end-to-end network, termed HyLFM-A-Net, which imposes channel attention^48^ on the existing HyLFM-Net, to further increase the computational efficiency of 3D reconstruction for VsLFM. HyLFM-A-Net is designed to accommodate high-resolution angular views with 3 × 3 scanning as input, with full-sampled high-resolution volumes as labels. The detailed architecture of HyLFM-A-Net is shown in Supplementary Fig. 17a,b. The output of Vs-Net prediction was used directly for the input of HyLFM-A-Net, with a patch size of 120 × 120 × 169 pixels (height × width × angle), while the target data consisted of the reconstruction results by iterative tomography with the size of 520 × 520 × 101 pixels (height × width × depth). 2D convolutions with channel attention were followed to extract features with a size of 120 × 120 × 64 pixels (height × width × channel), then two subpixel convolutions were used to recover the spatial resolution into 480 × 480 × 64 pixels. Another two convolutions with channel attention were used to adjust the feature channels into the size of 808, which was eightfold the output depth. We rearranged the 2D features into 3D features with a size of 480 × 480 × 8 × 101 pixels (height × width × channel × depth) and used 3D convolutions to fuse the features into 480 × 480 × 101 pixels (height × width × depth). A bicubic interpolation layer was attached to the end of the network to match the volume size of 520 × 520 × 101 pixels for supervision. A detailed comparison of HyLFM-Net and HyLFM-A-Net is given in Supplementary Fig. 17f. The channel size and feature size of HyLFM-Net and HyLFM-A-Net had been adjusted to our LFM set-up. HyLFM-A-Net follows the concept of HyLFM-Net, in which the depth dimension is rearranged using 2D channels that are integer multiples of the depth, and then the multiple is considered as the channel of 3D features. Attention operators were applied on 2D layers before rearrangement. For HyLFM-Net, the affine layer was used when it was trained with light-sheet continuous supervision, but when trained with sLFM reconstruction, the affine layer was removed.

We trained 400 epochs in 14 h on a single NVIDIA RTX 3090 GPU for convergence. During inference, the Vs-Net output with the size of 459 × 459 × 169 pixels was cropped into four overlapping patches with a size of 237 × 237 × 169 pixels, and the same sigmoid-based image fusion mentioned above was used for volume stitching. The whole inference time of HyLFM-A-Net for a single volume with a size of 1,989 × 1,989 × 101 pixels is approximately 6 s, while 11 s in total is required with Vs-Net inference involved, which is comparable to the inference time of HyLFM-Net. HyLFM-A-Net achieves a similar performance to that of 3D deconvolution in the imaging of living cells, with reduced computation costs at the cost of aberration robustness (Supplementary Fig. 17c–e). All of the deep learning networks used for comparison were trained on the same dataset as that used in VsLFM.

### Beads preparation and resolution characterization

For the fluorescent beads preparation, 1 ml 100-nm-diameter fluorescent beads (Thermo Fisher TetraSpeck Microspheres, T7279) were diluted with 100 ml pure water at room temperature to produce diluted fluorescent beads. Then 10 mg ml^−1^ diluted agarose was produced by mixing 1,000 mg pure agarose (Thermo Fisher UltraPure Low Melting Point Agarose, 16520100) with 100 ml pure water at 80 °C. When diluted agarose cooled to 40 °C, 1 ml diluted agarose and 1 μl diluted fluorescent beads were mixed well. Next, a 200 μl mixture of beads and agarose was put into a 35 mm dish (Thermo Fisher Nunc glass bottom dish, 150682) and left for 30 min to solidify to produce a uniform 3D distribution of beads. A ×63/1.4 NA oil-immersion objective was selected to verify the high-resolution capability of VsLFM. The temperature of the imaging environment was controlled at around 27 °C. For quantitative resolution analysis, the FWHM was calculated by measuring the intensity distributions of the reconstructed cross-section planes of the beads laterally and axially using a Gaussian fit. The calculation was conducted with MATLAB software on the results of LFM, VsLFM and sLFM, respectively. The FWHMs are presented as bar plots, in which the mean values and the standard deviations indicate the distribution of spatial resolution at different axial positions.

### Living L929 cell imaging

L929 cells were cultured in DMEM (Gibco) medium supplemented with 10% FBS (Biological Industries), 2 mM GlutaMAX and 100 U ml^−1^ penicillin–streptomycin in 5% CO_2_ at 37 °C. The PiggyBac Transposon Vector System and Vigofect were used for cell transfection to generate L929 TSPAN4-mCherry and TOM20-GFP stable cell lines^54^. L929 cells were cultured on a fibronectin-coated confocal dish and in DMEM (no phenol red) (Gibco) medium for imaging. During imaging, a microscope incubator system (Tokai Hit, INUF-IX3D-F1) was used to maintain the environmental conditions of 37 °C and a CO_2_ concentration of 5%.

### Zebrafish imaging

For imaging of zebrafish embryos, the cultured embryos were injected with 300 pg *Tspan4a*-EGFP messenger RNA (synthesized in vitro with mMessage mMachine T7 kit, Ambion, AM1344) in one cell at the 16-cell stage. Then the embryos were mounted in 1% low-melting-point agarose. During imaging, a ×63/1.4 NA oil-immersion objective was used, and the environment temperature was set at around 27 °C. For imaging of blood flow dynamics in zebrafish larvae, *Tg*(*flk:EGFP*; *gata1:DsRed*) transgenic zebrafish embryos were collected and cultured in Holtfreter’s solution at 28.5 °C. At 3–4 days postfertilization the zebrafish larvae were anesthetized using ethyl 3-aminobenzoate methanesulfonate salt (100 mg l^−1^) and embedded in 1% low-melting-point agarose in 35 mm confocal dishes (Thermo Fisher Nunc glass bottom dish, 150682) for in vivo imaging. During imaging, a ×20/0.5 NA air-immersion objective was selected to cover an FOV of 600 × 600 μm^2^, and the environmental temperature was set at around 27 °C.

### Mouse experiments

The mice used in this project were male and wild type (C57BL6/J, around 7–8 weeks). Mice were housed with food and water available ad libitum under a 12 h light–dark cycle at 22 °C with a relative air humidity of ~50%. For the mice labeled with neutrophils and vessels, 1 μg Ly6G/Ly6C monoclonal antibody (PE-Cyanine7, eBioscience, 25-4317-82), 3 μg AF647-WGA (Alexa Fluor 647 Conjugate, Thermo Fisher, P21462) and 100 μl PBS were injected i.v. After 30 min, Avertin (350 mg kg^−1^) was injected i.p. into the mice for anesthetization. After 20 min, the deeply anesthetized mice were dissected to expose the living liver on a home-made holder with a 170-μm-thick coverslip for intravital imaging. A ×63/1.4 NA oil-immersion objective was selected to capture subcellular dynamics. During the intravital imaging, a 37 °C body temperature maintenance instrument (ThermoStar Homeothermic Monitoring System, RWD) was launched to maintain the mouse in the native physiological state.

### *Drosophila* experiments

*Drosophila* strains (*MB065B-GAL4* > *20×UAS-pAce*) were provided by the Schnitzer laboratory at Stanford University and the Zhong laboratory at Tsinghua University. The *Drosophila* used in our experiments were female at 3–7 days and raised on the standard cornmeal agar media with a 12 h light–dark cycle at 25 °C. Based on the protocol described previously^55^, *Drosophila* were anesthetized on ice and mounted on a 3D-printed plastic disk with free movement of the legs. Then, the posterior head capsule was opened using sharp forceps (5SF, Dumont) in carbonated (95% O_2_, 5% CO_2_) buffer solution with a pH of 7.3 and an osmolarity of 275 mosM. Next, the air sacs, tracheas and M16 muscle were removed to minimize brain movement^56^. Ultraviolet glue was also added around the proboscis. After the surgery, *Drosophila* were placed under the objective lens for imaging of voltage transients in sparsely labeled neurons. For neural recording of the response to odor stimulus, 3% benzaldehyde was fed in a 5 s on–5 s off cycle.

### Neural extraction and analysis

For neural analysis of *Drosophila* data, we manually selected several regions of interest (ROIs) as shown in Fig. 6. The temporal traces of neural activity were calculated as *ΔF/F*_*0*_ = (*F–F*_*0*_)*/F*_*0*_, where *F* is the averaged intensity of the ROI and *F*_*0*_ is the baseline intensity. *F*_*0*_ was calculated as the mean fluorescence in the ROI averaged over the entire time series. The neural spikes were identified as the local peaks that rose above a threshold value (2% for VsLFM results and 1.2% for sLFM results) after the median-filtered (40 ms window) version was subtracted from the *ΔF/F*_*0*_ curve, and visualized as flashes of light in Supplementary Video 6. Each identified spike was temporally aligned to the time at which its peak value of *ΔF/F*_*0*_ occurred, to generate the average spike waveforms. The FWHM of each spike was calculated by measuring the intensity distribution of the spike temporal trace using a Gaussian fit. The amplitude of each spike was calculated as the absolute value of the peak. The peak time map was extracted from a single peak in the captured movie, in line with the previously reported method^57^. Specifically, we first applied a spatial Gaussian filter with a standard deviation of 9 voxels and a wavelet-based denoising method to each voxel independently. Then we fitted the filtered data with a quadratic spline interpolation. Finally, we used the threshold crossing time on the rising edge as the peak time of a specific voxel to generate the whole peak time map. The video of subframe propagation was made based on the peak time map. The firing rate was calculated as the number of spikes per second in the resting or stimulated state, and the curve was obtained with a 1 s temporal sliding window.

### Ethics statement

This work complies with all relevant ethics regulations for animal research and testing. All experimental procedures were performed with ethics approval from the Animal Care and Use Committee (IACUC) of Tsinghua University.

### Data analysis

All data processing and analyses were performed with customized MATLAB (MathWorks, MATLAB 2018b) scripts and Python (v3.7) scripts. The data collection and hardware control were performed with LABVIEW (2019 version) and our previously developed graphical user interface (sLFdriver^45^, v2.0). The 3D volumes of *Drosophila* brain in Fig. 6 were rendered using Imaris (v9.0.1). The 3D rendering of the volumes in the supplementary videos was carried out using Voltex modules in Amira (Thermo Fisher Scientific, Amira 2019). The 3D tracking of blood cells in the vessels of the zebrafish larvae and the neutrophil in the vessels of mouse liver was carried out automatically using Imaris (v9.0.1).

### Performance metrics

We chose signal-to-noise ratio, structural similarity (SSIM) and cut-off frequency (*k*_*c*_)^58^ to quantitatively evaluate the capability of VsLFM. Synthetic volumes or sLFM results were regarded as ground truth, as described in the Figure legends. The signal-to-noise ratio is calculated by the following formula:

$${\mathrm{SNR}} = 10\log _{10}\frac{{\left\| X \right\|_2^2}}{{\left\| {X - Y} \right\|_2^2}},$$

where *X* represents the ground truth and *Y* represents the corresponding reconstructed results. SSIM is calculated by the following formula:

$${\mathrm{SSIM}} = \frac{{\left( {2{\mu} _{X}{\mu} _{Y} + \left( {0.01 \cdot L} \right)^2} \right)\left({2{\sigma}_{XY} + \left( {0.03 \cdot L} \right)^2} \right)}}{{\left( {\mu _X^2 + \mu _Y^2 + \left( {0.01 \cdot L} \right)^2} \right)\left( {\sigma _X^2 + \sigma _Y^2 + \left( {0.03 \cdot L} \right)^2} \right)}},$$

where *X* and *Y* represent the signals, *µ*_*X*_ and *µ*_*Y*_ represent the average values of each signal, *σ*_*X*_ and *σ*_*Y*_ represent the corresponding standard deviations of each signal, and *σ*_*XY*_ represents the cross-covariance for *X* and *Y*. The dynamic-range value *L* in this work is 1 after the data were normalized to single-precision floating-point numbers. The SSIM indices were calculated on 2D images (Supplementary Figs. 3h, 4b, 8b, 8e, 9d, 18d) or 3D images (Fig. 3f and Supplementary Figs. 4a, 19b), according to different evaluation requirements. For SSIM calculation on 2D images, we calculated the local SSIM maps with multiple (sliding) 2D local Gaussian windows (with the size of 11 × 11 and standard deviation of 1.5) and averaged them to produce the SSIM metric. For SSIM calculation on 3D images, we first reshaped the 4D angular views (height × width × 13 × 13) into a 3D form (height × width × 169). The 169 angles were arranged based on their spatial proximity. Then local SSIM maps were computed in multiple (sliding) 3D local Gaussian windows (with the size of 11 × 11 × 11 and standard deviation of 1.5) of the 3D image. Finally, the mean value of the local SSIM maps was returned as the SSIM metric. In practice, the calculations were conducted using the built-in ssim.m function in MATLAB R2018b. For decorrelation analysis of images^58^, the cross-correlation coefficient is calculated by the following formula:

$$d\left( r \right) = \frac{{{\int} {{\Re} \left\{ {I\left( {{{\boldsymbol{k}}}} \right)I_n^ \ast \left( {{{\boldsymbol{k}}}} \right)M\left( {{{{\boldsymbol{k}}}};r} \right)} \right\}dk_xdk_y} }}{{\sqrt {{\int} {\left| {I\left( {{{\boldsymbol{k}}}} \right)} \right|^2} dk_xdk_y{\int} {\left| {I_n\left( {{{\boldsymbol{k}}}} \right)M\left( {{{{\boldsymbol{k}}}};r} \right)} \right|^2dk_xdk_y} } }},$$

where *I*(***k***) denotes the Fourier transform of the input image, *I*_*n*_(***k***) denotes the normalized form of *I*(***k***), ***k*** = (*k*_*x*_, *k*_*y*_) denotes the Fourier space coordinates and *M*(***k****;r*) denotes the binary mask of radius *r*. Then the cut-off frequency (*k*_*c*_) is calculated by the following formula:

$$k_c = \max \left\{ {r_i} \right\},$$

where *r*_*i*_ denotes the radius of the binary mask, corresponding to the frequency of the highest peak. We also used the Pearson correlation coefficient (*R*) to evaluate the similarity between the ground truth and the results by different methods. *R* is calculated by the following formula:

$$R = \frac{{E\left[ {\left( {X - \mu _X} \right)\left( {Y - \mu _Y} \right)} \right]}}{{\sigma _X\sigma _Y}},$$

where *X* and *Y* denote the signals, *µ*_*X*_ and *µ*_*Y*_ represent mean values of each signal, *σ*_*X*_ and *σ*_*Y*_ denote the corresponding standard deviations of each signal, and *E*[·] denotes the expectation. For quantitative analysis on Fourier spectra (Fig. 4e), the Fourier ring correlation (FRC) were applied as described in the original paper^43^. In the calculation of the FRC curve, the threshold was set to 1/7 ≈ 0.143 for the cut-off frequency.

### Statistics and reproducibility

Biological data shown in Figs. 2, 4, 5, 6 and Supplementary Figs. 4, 5, 6, 15, 16 are representative of *n* = 6 experiments. Biological data shown in Supplementary Figs. 9 and 13 are representative of *n* = 12 experiments. Simulated data shown in Fig. 3 and Supplementary Figs. 10, 12 are representative of *n* = 17 experiments. Simulated data shown in Supplementary Figs. 1, 8, 11, 14, 17, 18, 19 are representative of *n* = 20 experiments.

### Reporting summary

Further information on research design is available in the Nature Portfolio Reporting Summary linked to this article.

## Online content

Any methods, additional references, Nature Portfolio reporting summaries, source data, extended data, supplementary information, acknowledgements, peer review information; details of author contributions and competing interests; and statements of data and code availability are available at 10.1038/s41592-023-01839-6.

## Supplementary information

Supplementary Information Supplementary Figs. 1–19, Supplementary Tables 1, 2 and titles of Supplementary Videos 1–6.

Reporting Summary

Supplementary Video 1 Long-term observation of living L929 cells to demonstrate the resolution improvements of VsLFM.

Supplementary Video 2 Experimental comparisons between LFM, VsLFM and sLFM on complicated 3D membrane dynamics in zebrafish embryos in vivo.

Supplementary Video 3 Highly dynamic circulating blood flow in a zebrafish larva at 50 vps to compare the generalization ability of VsLFM, VCD-Net and HyLFM-Net, which were all trained on mouse liver data.

Supplementary Video 4 VsLFM eliminates the motion artifacts of sLFM in living mouse livers with extremely high-speed motions during respiration.

Supplementary Video 5 Intravital subcellular imaging of neutrophil migration and retraction fiber formation by VsLFM with heart beat accompaniment in mouse liver.

Supplementary Video 6 3D imaging of voltage activities across the whole brain with subcellular resolution in Drosophila at ultrahigh speed of 500 vps, enabling the observation of 3D propagation of action potentials in a single neuron.

## Data Availability

The Bio-LFSR dataset includes more than 1,300 pairs of 4D low-resolution and high-resolution images, covering four species, six structures and multiple imaging conditions, and is made publicly accessible on Zenodo (10.5281/zenodo.7233421)^59^. Supporting data for Vs-Net have been made publicly available on GitHub (https://github.com/THU-IBCS/VsLFM-master/tree/main/Data).

## Code availability

All relevant codes of VsLFM are readily accessible and available on Github (https://github.com/THU-IBCS/VsLFM-master) and Zenodo (10.5281/zenodo.7436082)^60^.
