# Supplementary material for: Virtual-scanning light-field microscopy for robust snapshot high-resolution volumetric imaging
Source: Nat Methods. 2023 Apr 6;20(5):735–46. doi: 10.1038/s41592-023-01839-6 (PMC10172145; doi:10.1038/s41592-023-01839-6)
Supplement: Supplementary file 1 — Supplementary Figs. 1–19, Supplementary Tables 1, 2 and titles of Supplementary Videos 1–6. [file 41592_2023_1839_MOESM1_ESM.pdf]

nature methods

Article https://doi.org/10.1038/s41592-023-01839-6

Virtual-scanning light-field microscopy for robust snapshot high-resolution volumetric imaging

In the format provided by the authors and unedited

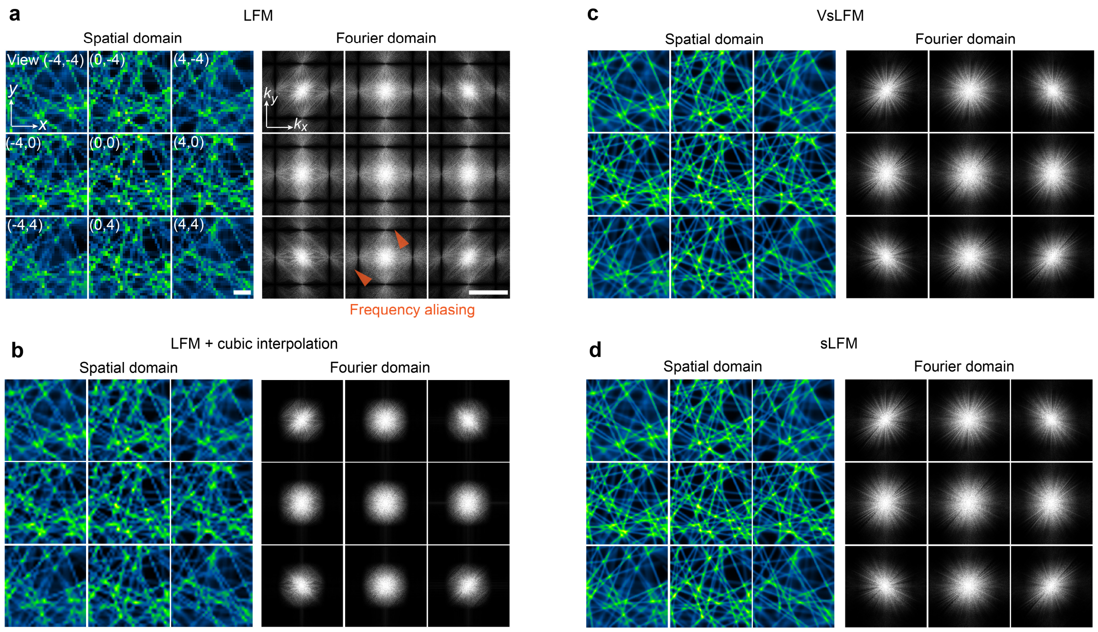

Supplementary Figure 1 | Spatial and Fourier domains of multi-view measurements by different methods. a-d 9 typical spatial angular views (left) and corresponding Fourier spectrums (right) of 1-μm-diameter synthetic tubulins obtained by LFM, LFM with cubic interpolation, VsLFM and sLFM, respectively. The high-frequency components of LFM is difficult to be precisely recovered by a simple cubic interpolation (**a-b**). On the contrary, VsLFM that learns the physical prior embedded between multiple phase-correlated angular measurements, can retrieve high-fidelity frequencies in the full range (**c**). The sLFM results shown in **d** serve as the reference, indicating that VsLFM keeps high consistency with sLFM both in spatial and frequency domains. Note that, this figure is extended from Fig. 1a for further analysis. Scale bars, 10 μm spatially and 1 μm^{-1} spectrally.

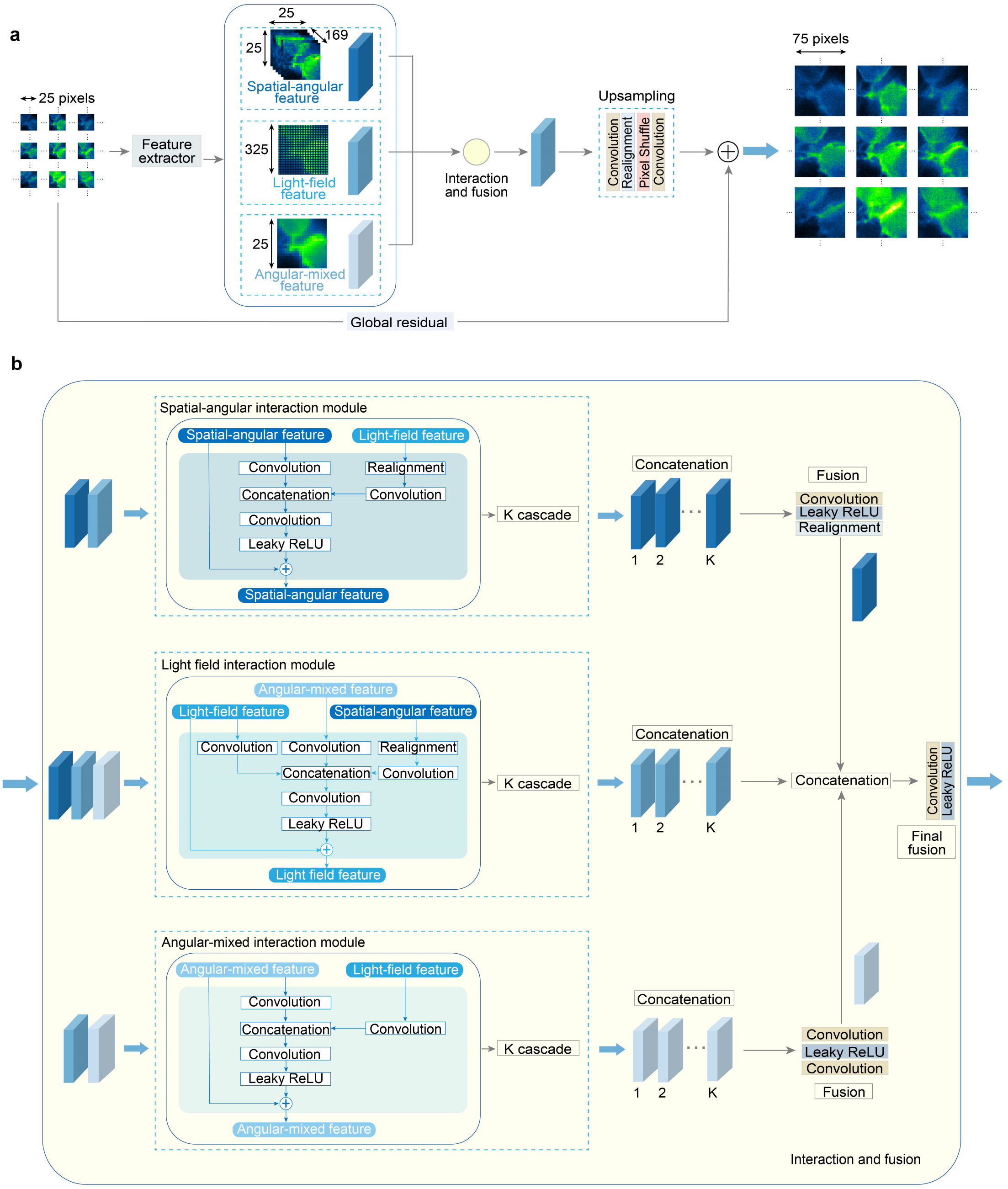

Supplementary Figure 2 | Overview of Vs-Net network architecture. a, Basic structure of the Vs-Net framework, which is a global residual network mainly containing three core components: the feature extraction, feature interaction and fusion, and feature upsampling. A feature extractor is firstly imposed to yield three types of features from low-resolution spatial-angular views, including the spatial-angular feature, the light-field feature that is transformed into the light-field domain through a pixel-realignment operator, and the angular-mixed feature that is weighted by different spatial-angular views with weighting coefficients continuously optimized during the network training. Then, high-resolution spatial-angular views are gradually generated through feature interaction and fusion, and feature upsampling in the network. The pixel size of each image is indicated by the numerical values next to it. **b**, Unfolded flowchart of the feature interaction and fusion, containing spatial-angular interaction module, light-field interaction module, angular-mixed

interaction module, and concatenation and fusion module. The operators such as convolution, leakyReLU and realignment are marked with boxes in the illustration. Among them, pixel realignment is a common operator in light-field imaging, used for the mapping between light-field domain and spatial-angular domain. Detailed network parameters of each layer are listed in Supplementary Table 1.

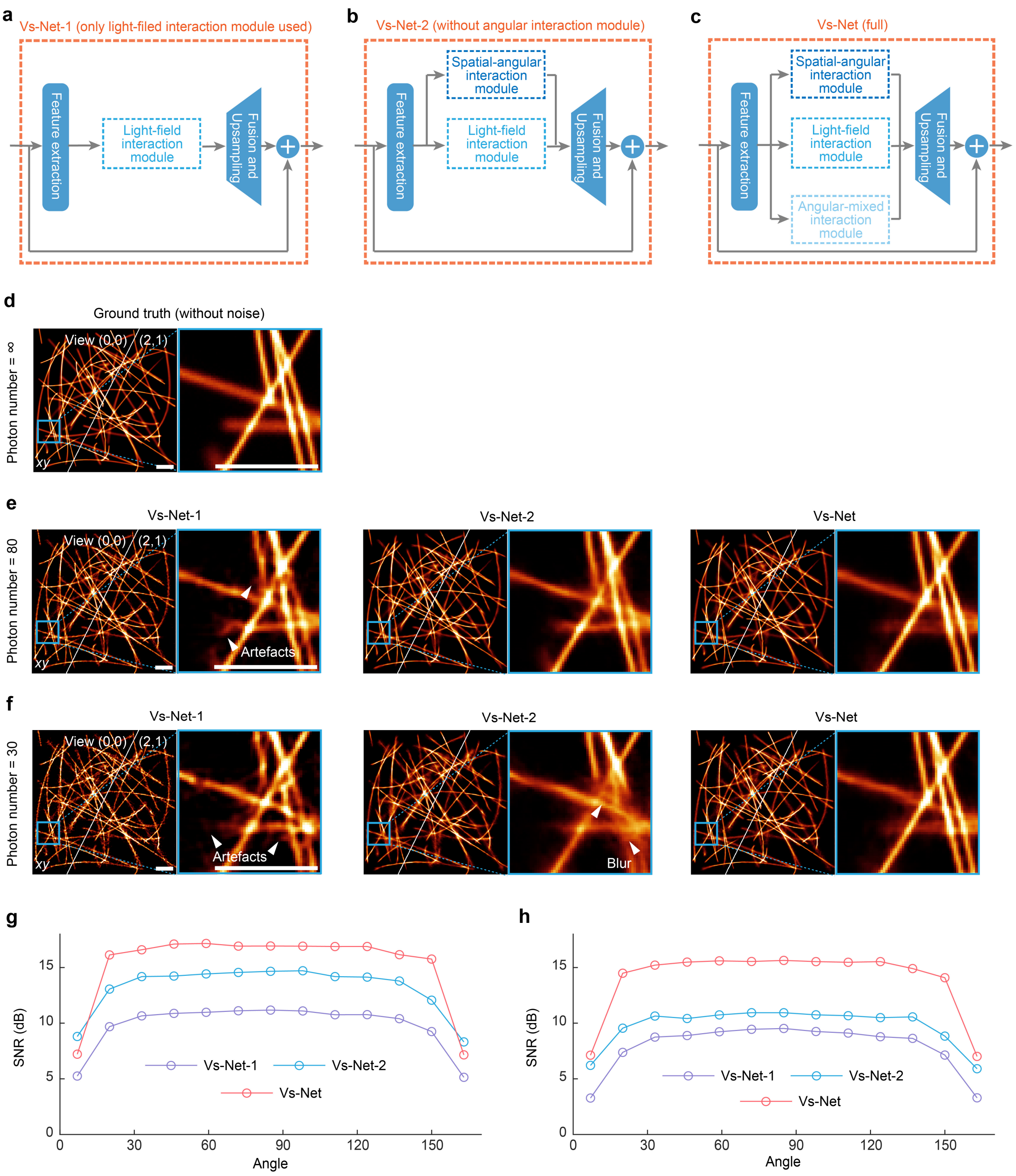

Supplementary Figure 3 | Ablation study on Vs-Net. a, Simplified schematic of Vs-Net-1, with only the light-filed interaction module used. **b**, Simplified schematic of Vs-Net-2, with light-filed interaction module and spatial-angular interaction module used. **c**, Simplified schematic of Vs-Net, with all three interaction modules used. **d**, The simulated ground truth of two spatial-angular views and enlarged views of 1-μm-diameter synthetic tubulins obtained by sLFM with a 63×/1.4NA oil-immersion objective in ideal imaging conditions. **e**-**f**, Corresponding output results obtained by Vs-Net-1 (left), Vs-Net-2 (middle) and Vs-Net (right) with the input contaminated by mixed Poisson-Gaussian noises. We set the image bit depth as 16, the variance of Gaussian noise as 5, and the photon number of the maximum intensity as 100 (**e**) or 10 (**f**) for the simulation. **g**-**h**, The SNR curves versus different angles of Vs-Net-1, Vs-Net-2 and Vs-Net with the maximum photon number of 100 (**g**) and 10 (**h**). The sLFM results serve as the ground truth for SNR calculation. Detailed adjustment of network structures is described in Methods. Scale bars, 20 μm (**d**-**f**).

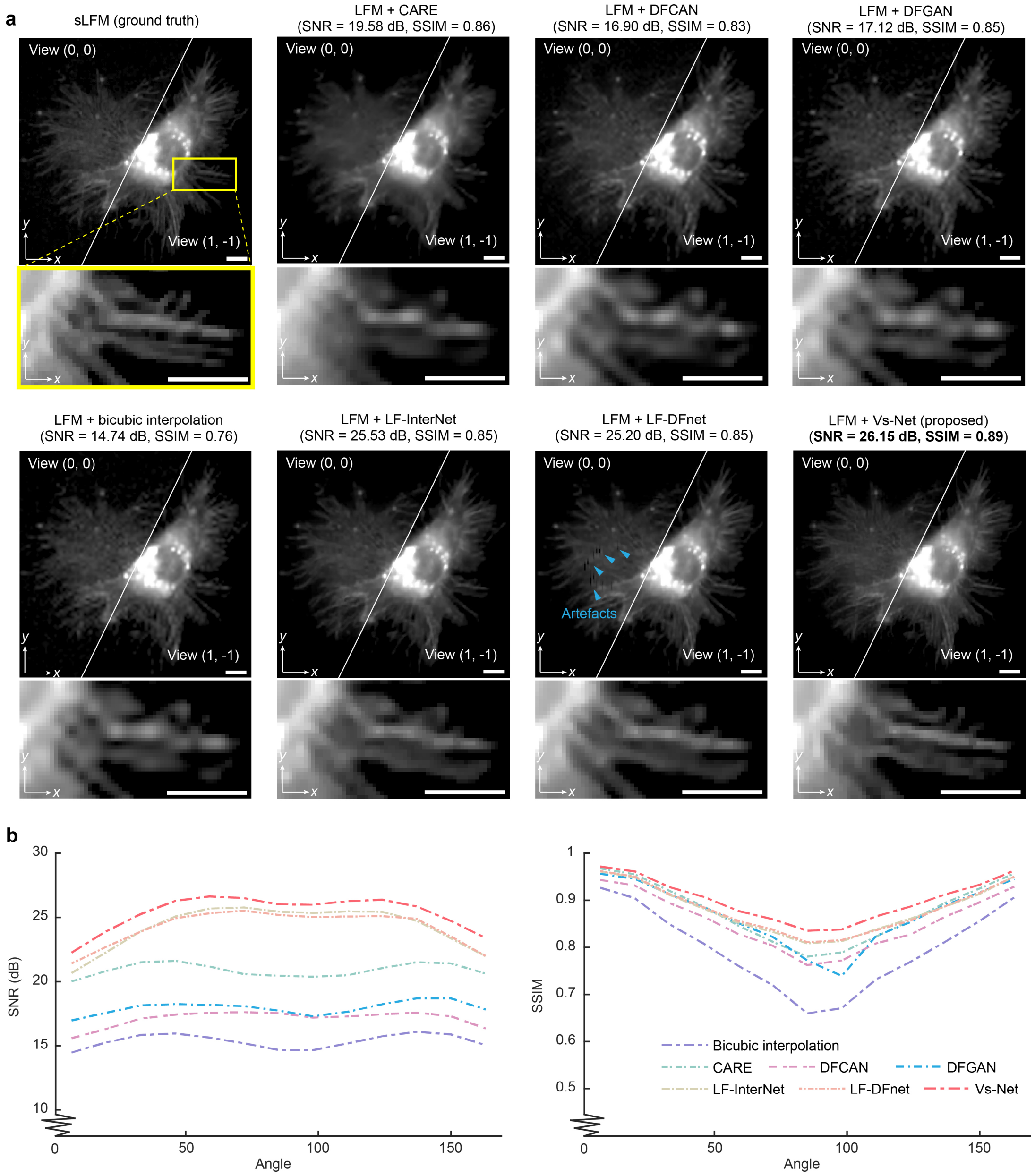

Supplementary Figure 4 | Comparisons between Vs-Net and state-of-the-art learning-based methods on spatial-angular measurements before reconstruction. a, Two representative high-resolution spatial-angular views of the same L929 cell with membrane labelling (TSPAN4-mCherry) as demonstrated in Fig. 2a, obtained by different methods. We used sLFM as the ground truth for comparison. The other results were obtained by LFM after processing with bicubic interpolation, CARE, DFCAN, DFGAN, LF-InterNet, LF-DFnet and Vs-Net, respectively. The SNR and SSIM indices marked in the top were calculated based on all 13 × 13 angular views. For fair comparisons, the training parameters including data pairs, training epoch were set almost the same, which concurrently ensured that all learning-based algorithms reached convergence during the training process (Methods). **b**, The curves of the SNR and SSIM versus different angles separately, indicating the superior performance of Vs-Net over state-of-the-art learning-based methods. Scale bars, 10 μm.

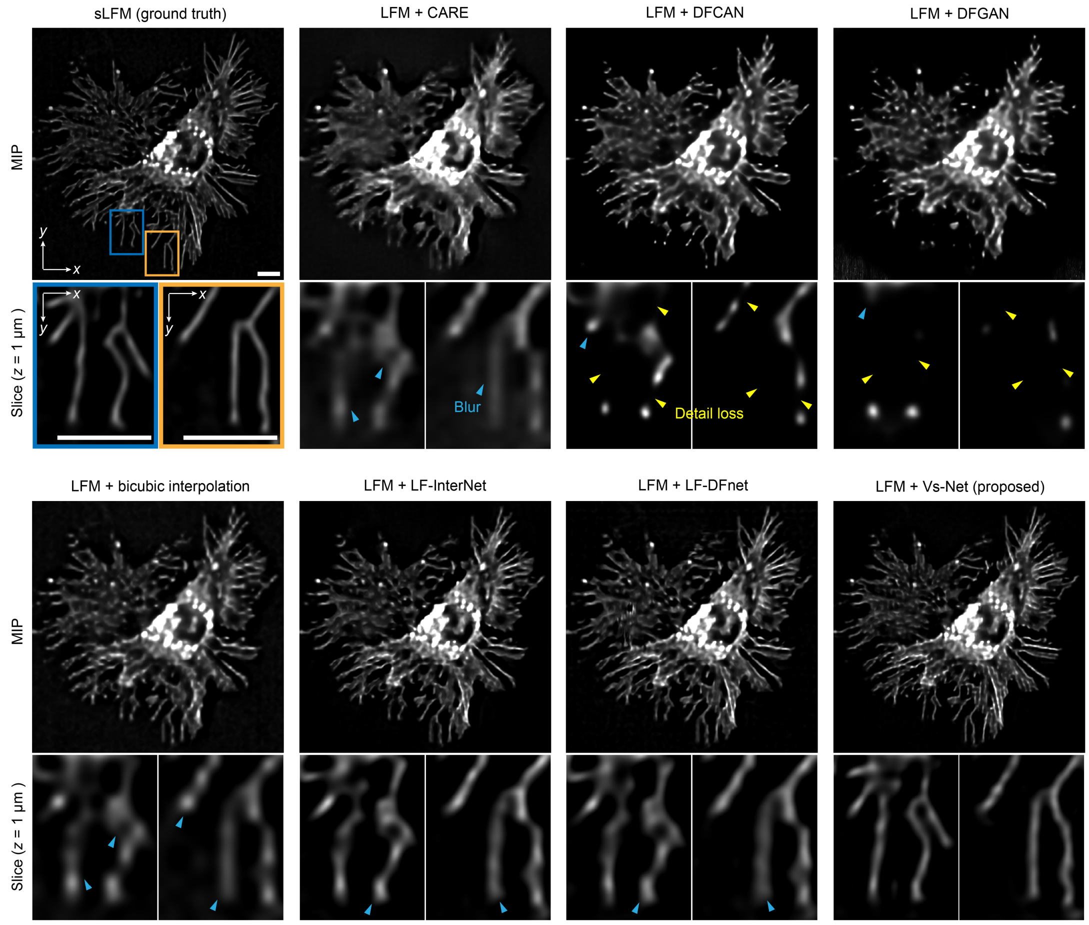

Supplementary Figure 5 | Comparisons of the reconstructed volumes between the proposed Vs-Net and state-of-the-art SR algorithms applied on spatial-angular measurements. MIPs and two selected axial slices of reconstructed results of the spatial-angular views in Supplementary Fig. 4 by different methods were compared. The sLFM results were used as ground truth. The arrows indicate the blur and artefacts. Scale bars, 10 μm.

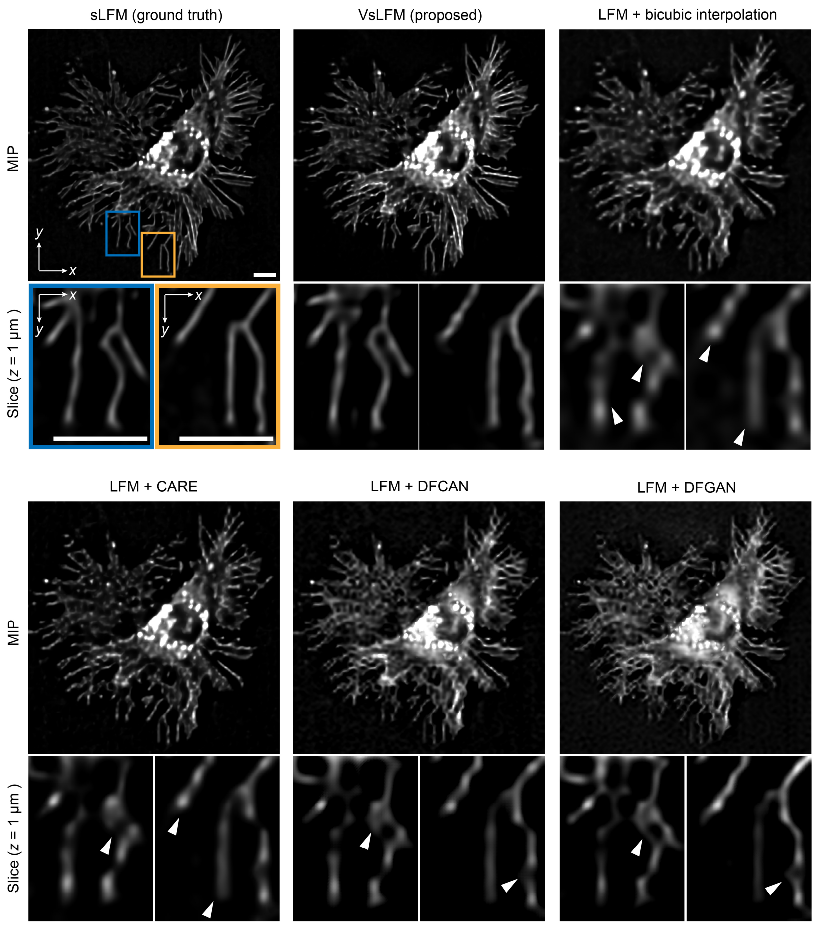

Supplementary Figure 6 | Comparisons between the reconstructed volumes of our VsLFM and the results of SISR algorithms applied on the reconstructed volumes of LFM. MIPs and two selected axial slices of reconstructed results by sLFM, VsLFM, LFM, CARE, DFCAN and DFGAN, respectively. The results of sLFM, VsLFM and LFM are consistent with those in Supplementary Fig. 5. In this figure, CARE, DFCAN and DFGAN were trained using reconstruction results of LFM as input and corresponding reconstruction results of sLFM as targets. The training dataset is the same as that used in Supplementary Fig. 4. The training of CARE network was performed on 3D volume pairs, while DFCAN and DFGAN were trained on 2D slice pairs segmented from 3D volumes. For fair comparisons, all learning-based algorithms reached convergence during the training process, with detailed training parameters including data pairs, training epoch, processing time and system environments described in Methods. The sLFM results served as ground truth. The white arrows indicate the blur and detail loss. Scale bars, 10 μm.

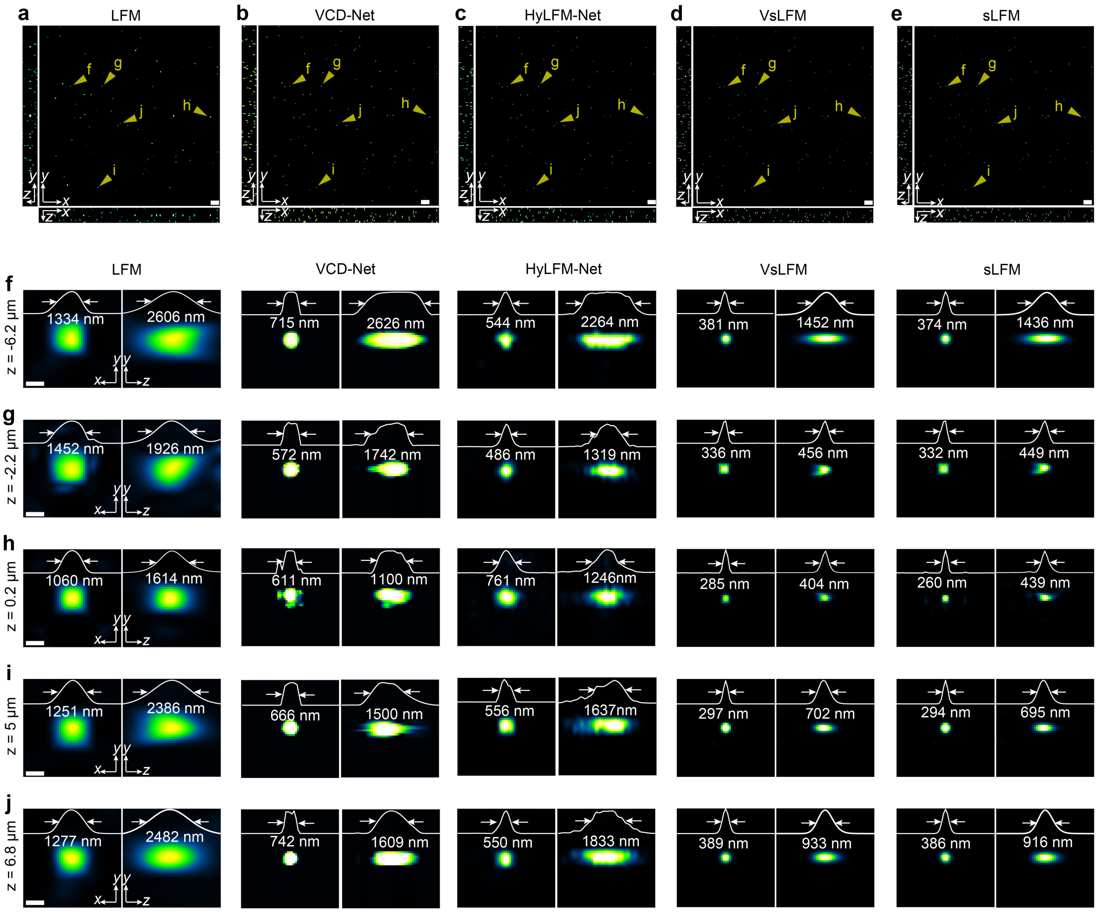

Supplementary Figure 7 | Comparisons of resolution at different axial positions among LFM, VCD-Net, HyLFM-Net, VsLFM and sLFM. a-e, Orthogonal MIPs of 100-nm fluorescence beads uniformly distributed in low-melt agarose, obtained by different methods with a 63×/1.4NA oil-immersion objective. **f-j**, Enlarged MIPs of individual reconstructed beads at different axial positions (*z* = -6.2 µm, -2.2 µm, 0.2 µm, 5 µm and 6.8 µm). The selected beads are marked by yellow arrows in **a-e**. The lateral and axial intensity profiles and FWHMs were plotted inside the MIP images, demonstrating that the resolution of VsLFM is close to that of sLFM at each depth and much better than the resolution of other methods. Scale bars, 10 µm (**a-e**) and 1 µm (**f-j**).

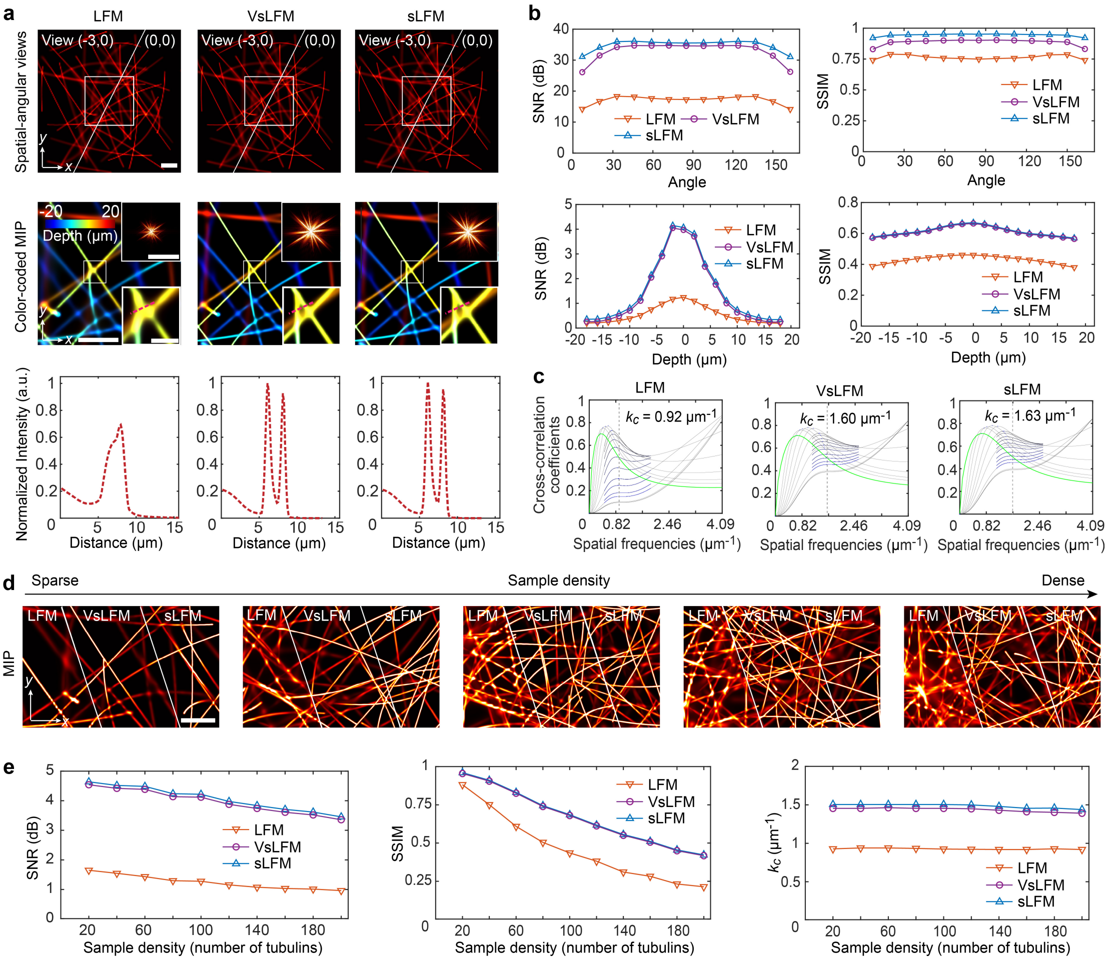

Supplementary Figure 8 | Quantitative evaluation of VsLFM imaging performance on synthetic tubulins with different sample densities. a, Two spatial-angular views of 1-μm-diameter synthetic tubulins obtained by LFM (left), VsLFM (middle) and sLFM with 3 × 3 scanning (right), are shown on the first row. Corresponding depth-coded reconstructed MIPs of enlarged regions, with insets displaying the Fourier spectrums and zoom-in views, are shown on the second row. Normalized intensity profiles along the marked red dashed lines are shown on the last row, demonstrating that VsLFM can resolve densely aliasing crisscrossing regions. The synthetic data were generated with random distributions. **b**, The curves of reconstructed SNR and SSIM versus different angles (top row) and reconstructed depths (bottom row) by LFM, VsLFM and sLFM. We used sLFM results with 13 × 13 full scanning as the ground truth for the calculation of SNR and SSIM in the evaluation along angles (top row), whereas the synthetic volume was used directly as ground truth in the evaluation along depths (bottom row). **c**, Decorrelation functions and the estimated cut-off frequency k_c of the *xy*-MIPs in **a** by different methods. Gray curves represent decorrelation functions with high-pass filtering; green curves represent the decorrelation function without any high-pass filtering; vertical gray dashed lines represent the cut-off frequency. **d**, *xy*-MIPs stitched by LFM, VsLFM, and sLFM results, with gradually increasing sample densities. **e**, The curves of SNR, SSIM and k_c versus sample densities for LFM, VsLFM and sLFM. The sample

density is defined by the number of tubulins in the whole field of view. Scale bars, 20 μm (**a**, **d**), 5 μm and 2 μm^{-1} (insets in **a**).

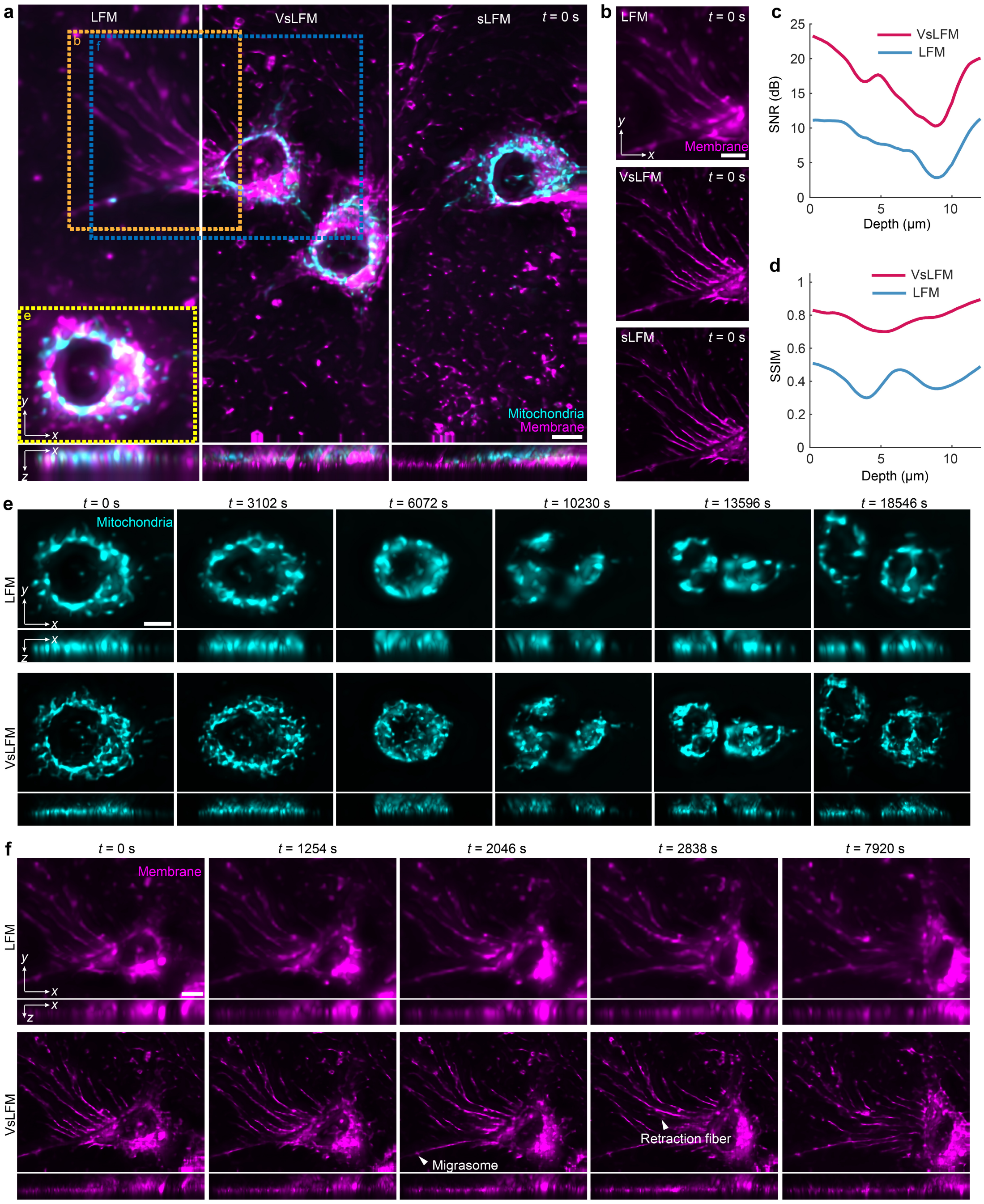

Supplementary Figure 9 | Long-term observation of living L929 cells with subcellular resolution and low phototoxicity. a, Orthogonal MIPs of living L929 cells with mitochondria (cyan) and membrane (magenta) labelling, obtained by LFM (left), VsLFM (middle) and sLFM (right) with a 63×/1.4NA oil-immersion objective at $t = 0$ s. **b**, MIPs of the selected region in **a** at $t = 0$ s, obtained by LFM, VsLFM and sLFM, respectively. Only membrane channel is shown for clear comparisons. **c-d**, SNR and SSIM indices across the depth range of LFM and VsLFM results in **b**, revealing superior performance of VsLFM. We used sLFM result as the reference for SNR and SSIM calculations. **e**, Mitochondria-channel orthogonal MIPs of the region marked in **a** at different time stamps. The whole imaging duration covers more than 5 hours continuously, without noticeable reduction in fluorescent intensity. The upper row shows the results of LFM, while the lower row

shows the results of VsLFM with higher resolution to distinguish the subcellular structures. **f**, Membrane-channel orthogonal MIPs of the region marked in **a** at different time stamps, obtained by LFM and VsLFM, respectively. Membrane dynamics such as fiber retractions and migrasome formations can be observed by VsLFM. Scale bars, 10 μm (**a-b**, **e-f**).

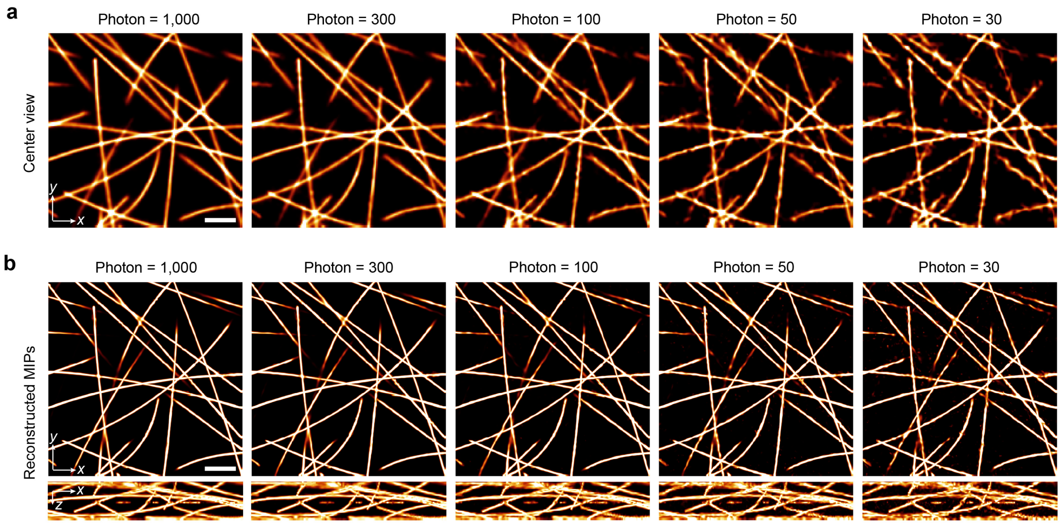

Supplementary Figure 10 | Evaluation of VsLFM imaging performance on synthetic tubulins at different noise levels. a, The high-resolution angular view (center view) of Vs-Net of the same synthetic tubulins as demonstrated in Fig. 3, with increasing shot noise levels indicated by the reduction of maximum photon number. Note that in this simulation, Vs-Net was only trained on synthetic tubulins without noise. **b**, The reconstructed MIPs of VsLFM at different noise levels, indicating the noise robustness of the method. Scale bars, 10 μm.

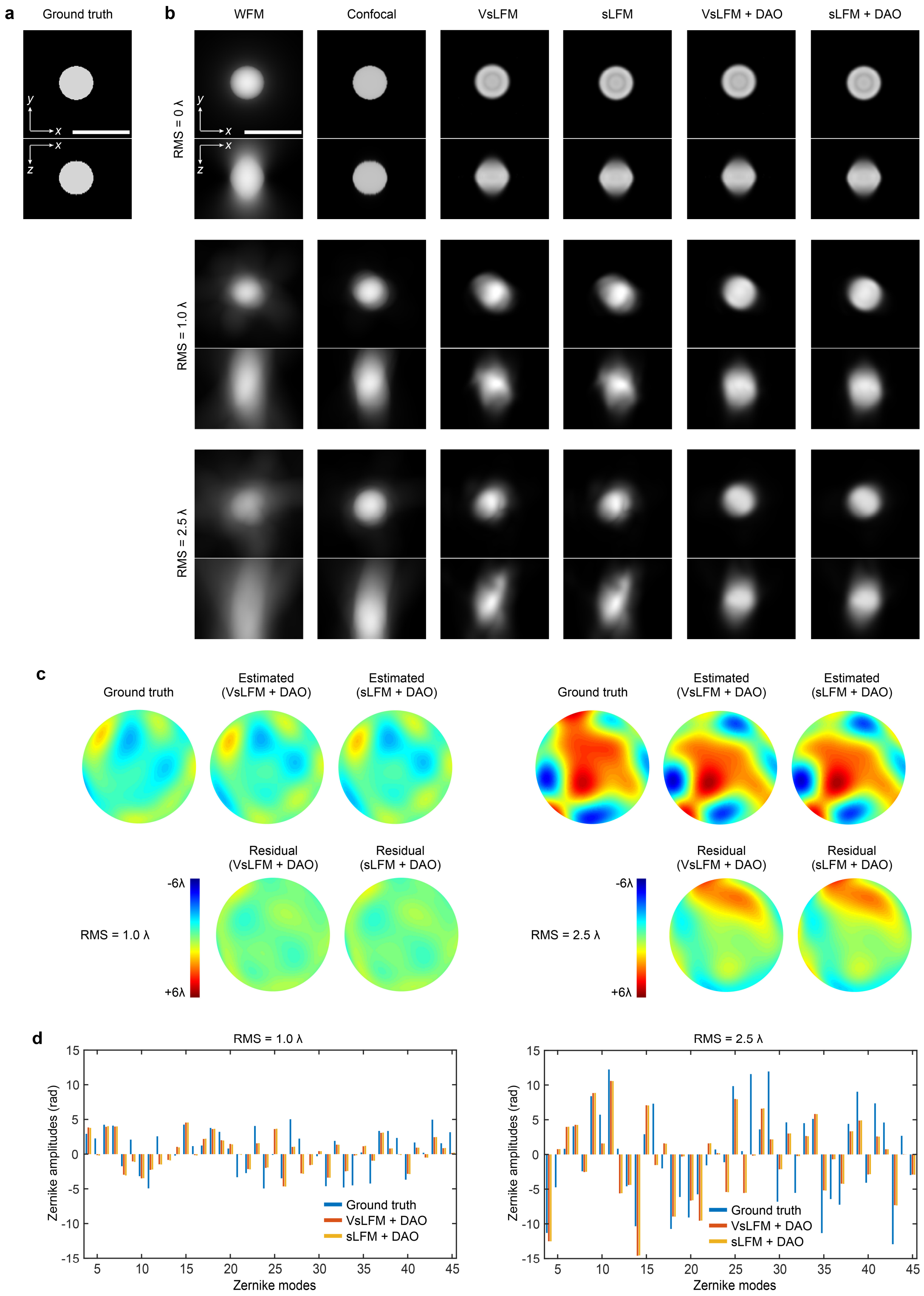

Supplementary Figure 11 | Quantitative evaluation of the DAO performance of VsLFM. a, Orthogonal MIPs of a synthetic 6-μm-diameter fluorescence bead as the ground truth for simulation. **b**, Simulated orthogonal MIPs of the beads obtained by WFM, confocal microscopy, VsLFM, sLFM, VsLFM with DAO and sLFM with DAO, respectively. Different rows correspond to imaging conditions of different aberration levels in terms of root mean square (RMS) of 0.0 λ, 1.0 λ, and 2.5 λ. The results reveal that VsLFM is compatible with DAO for aberration correction. **c**, The ground-

truth aberrations, estimated aberrations by VsLFM with DAO, estimated aberrations by sLFM with DAO and the corresponding error maps, under the given aberrations of 1.0 λ RMS (left) and 2.5 λ RMS (right). **d**, Comparisons of the amplitudes of different Zernike modes decomposed from the ground truth and estimated aberration wavefronts. Scale bars, 10 μm (**a-b**).

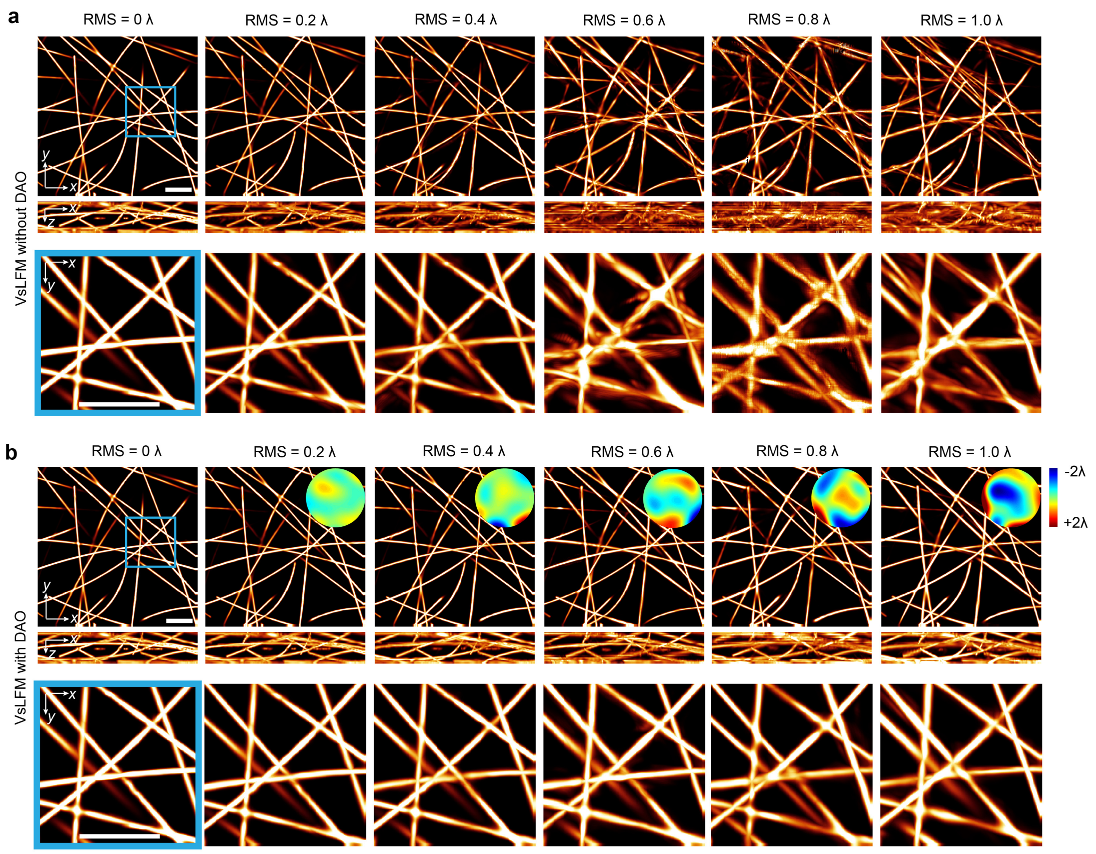

Supplementary Figure 12 | Evaluation of VsLFM with and without DAO on synthetic tubulins at different levels of optical aberrations. a-b, Orthogonal MIPs and enlarged views of the synthetic tubulins, obtained by VsLFM without DAO (**a**) and VsLFM with DAO (**b**) at increasing aberration levels. As the aberration level increases, distorted disparities gradually appear in angular measurements and lead to ghosting and obscures after reconstruction, which can be accurately corrected by DAO. The estimated wavefronts by DAO are shown in the insets of **b**. Note that in this simulation, Vs-Net was only trained on synthetic tubulins without aberration. Scale bar, 10 μm.

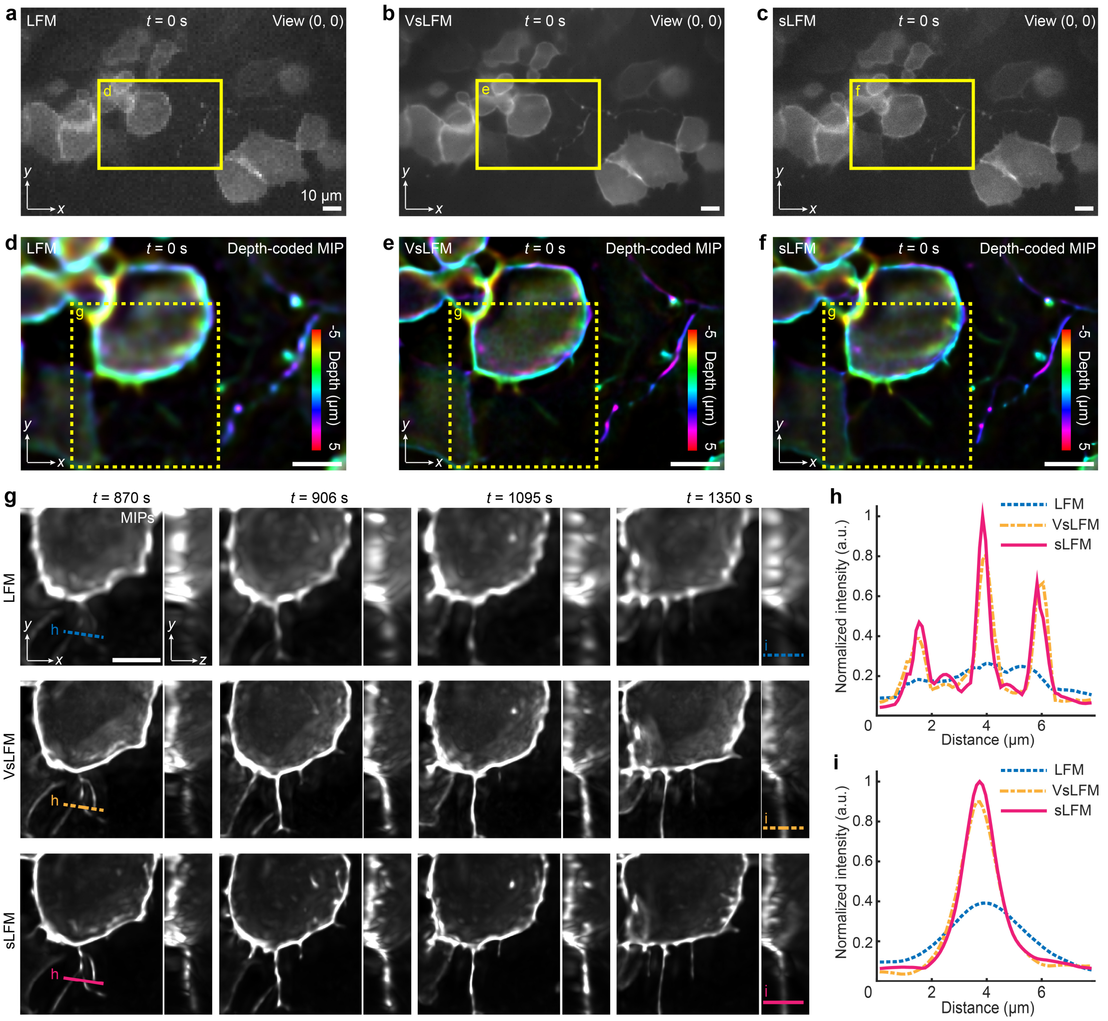

Supplementary Figure 13 | High-speed 3D *in vivo* imaging of subcellular membrane dynamics in zebrafish embryos. a-c, Center views obtained by LFM, VsLFM and sLFM at t = 0 s, respectively. The VsLFM results exhibit better noise suppression than sLFM, with the marginal amount of residual salt-and-pepper-like noises. **d-f**, Depth-coded MIPs of the corresponding zoom-in region at t = 0 s. Different colors correspond to different depths. The noise was significantly attenuated after reconstruction, since the 3D deconvolution process also combined multiple angular measurements for better SNR. **g**, Comparisons between LFM, VsLFM and sLFM on the dynamic retraction fibers. Orthogonal MIPs of the region marked in **d-f** by LFM, VsLFM and sLFM (from top to bottom) are displayed at different time stamps. **h**, Normalized lateral intensity profiles along the lines marked in **g**, demonstrating the lateral resolution improvement and contrast enhancement of VsLFM. The intensity profile of sLFM serves as ground truth, revealing that VsLFM has high fidelity for fine biological structures. **i**, Normalized axial intensity profiles along the lines marked in **g**, exhibiting that VsLFM has a significantly higher axial resolution than LFM. Scale bars, 10 μm.

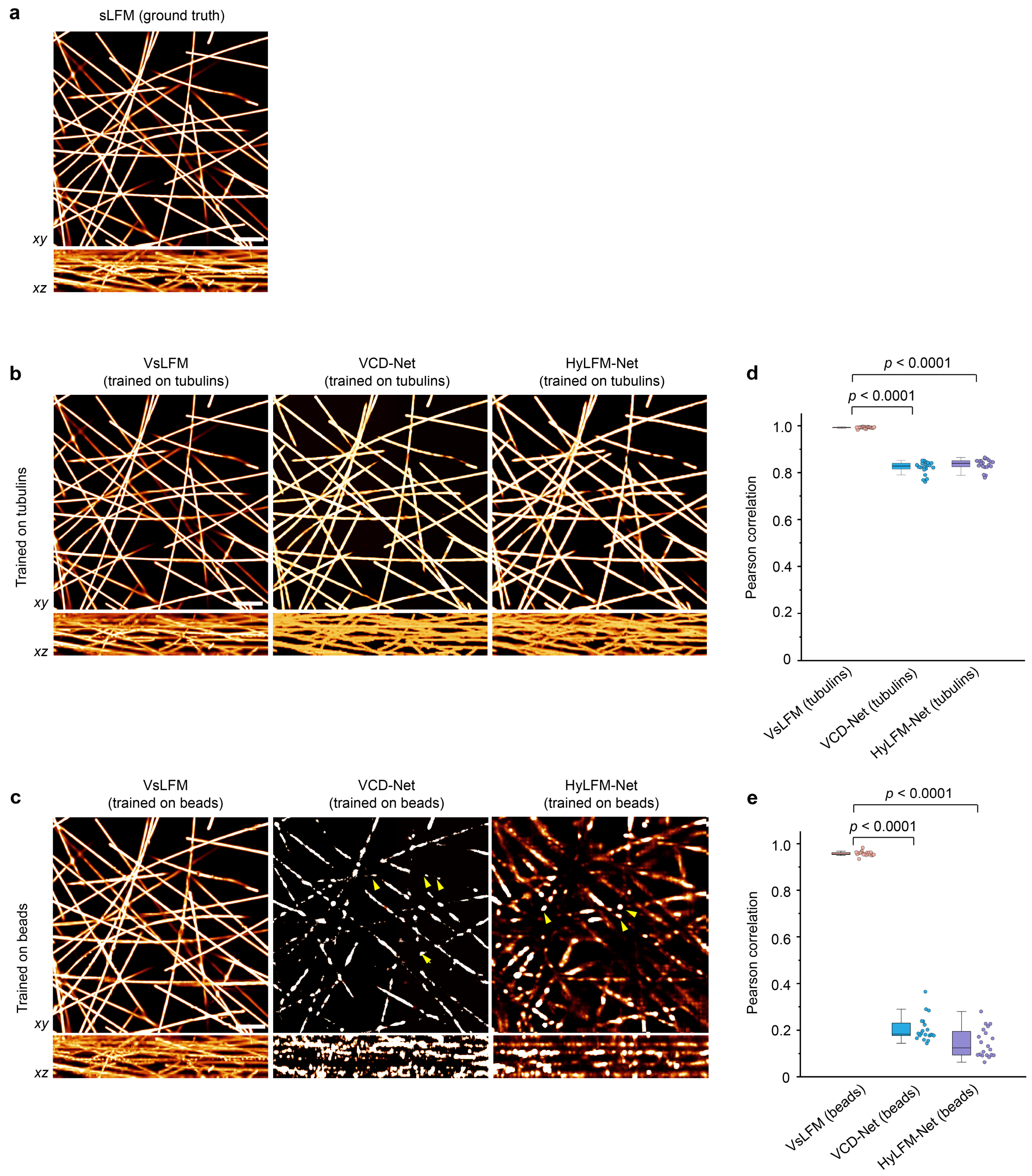

Supplementary Figure 14 | Quantitative comparisons of the generalization ability between VsLFM, VCD-Net and HyLFM-Net on synthetic data. a, Simulated ground-truth MIPs of 1-μm-diameter synthetic tubulins, reconstructed by sLFM with a 63×/1.4NA oil-immersion objective. **b**, Results obtained by VsLFM, VCD-Net and HyLFM-Net. All three networks were trained on the same tubulin dataset. **c**, Results obtained by VsLFM, VCD-Net and HyLFM-Net, trained on a bead dataset. Due to large morphological differences between beads and tubulins, the performance of end-to-end networks degraded quickly. A large number of structural fragments that resemble the shape of small beads appear in the results of VCD-Net and HyLFM-Net, while VsLFM remains stable performance. **d**, Boxplots showing Pearson correlations of results obtained by different methods trained on the tubulins dataset and tested on the tubulins. **e**, Boxplots showing Pearson correlations of results obtained by different methods trained on the beads dataset and tested on the tubulins. The results show that Vs-Net has much better generalization ability on diverse sample

structures with better stability than VCD-Net and HyLFM-Net. The format of boxplots in **d** and **e**: center line, median; box limits, lower and upper quartiles; whiskers, 1.5-fold interquartile range. P values were calculated by the two-sided paired t-test. In panel (**d**), $P = 4.15\times10^{-27}$ for VCD-Net and $P = 1.18\times10^{-27}$ for HyLFM-Net; in panel (**e**), $P = 8.36\times10^{-39}$ for VCD-Net and $P = 1.65\times10^{-39}$ for HyLFM-Net. $n = 20$ for each method. Different data points come from tubulins with different 3D distributions randomly. In this experiment, VCD-Net and HyLFM-Net were trained with the synthetic tubulins or beads as targets and corresponding light-field measurements as input. Scale bars, 10 μm.

Generalized VsLFM

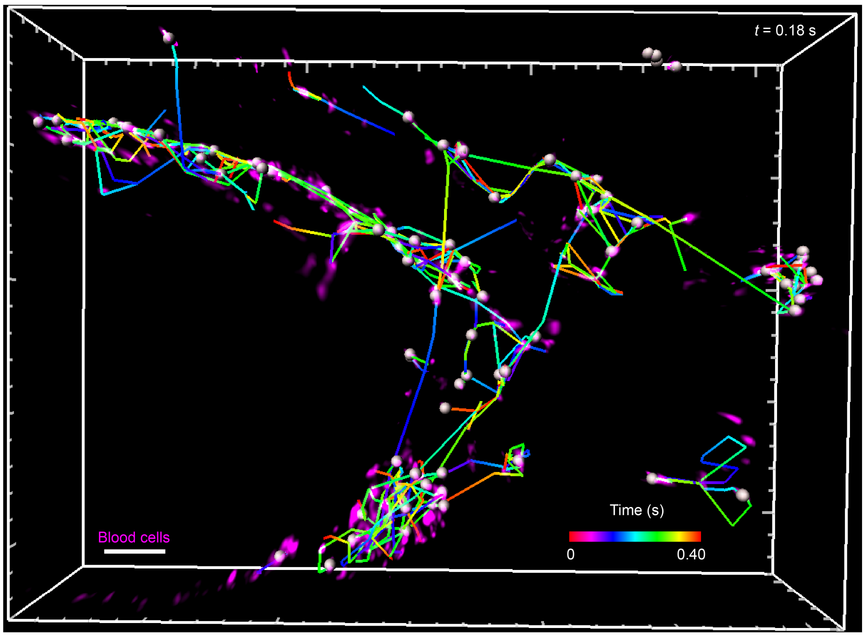

Supplementary Figure 15 | 3D tracking of blood cells in zebrafish larva with the VsLFM trained on the mouse data. The 3D tracking was accomplished automatically through Imaris 9.0.1 software, with the overall tracking time length of 0.40 s, from $t = 0$ s to $t = 0.40$ s. 76 flowing blood cells were identified by white spheres and tracked with temporal-coding trajectory. The color bar in the bottom right corner represents different time stamps. Scale bar, 50 μm.

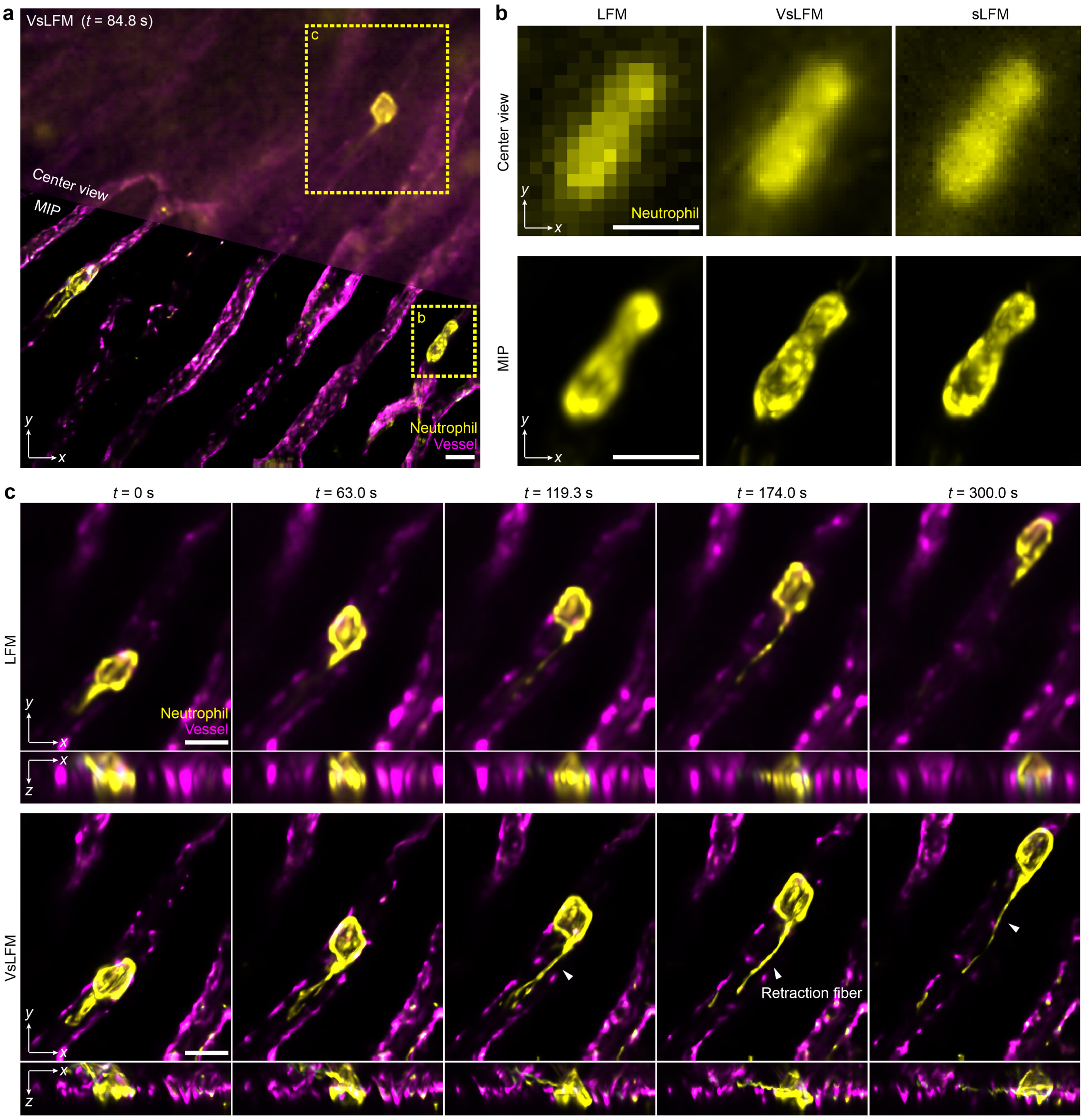

Supplementary Figure 16 | Intravital subcellular imaging of neutrophil migration and retraction fiber formation in mouse liver at 1.33 VPS. a, Center view (top) and corresponding MIP (bottom) of the neutrophils (yellow) and blood vessels (magenta) in a living mouse liver obtained by VsLFM. **b**, Enlarged center views (top) and MIPs (bottom) of the region marked in **a**, obtained by LFM (left), VsLFM (middle) and sLFM (right), respectively. **c**, Orthogonal MIPs at different time stamps, obtained by LFM (upper row) and VsLFM (lower row). A retraction fiber indicated by the white arrows was generated during neutrophil migration, which was hard to be resolved in LFM. The whole migration dynamics under strong vibrations due to respiration, is clearly observed by VsLFM without motion artefacts (Supplementary Video 5). Scale bar, 10 μm.

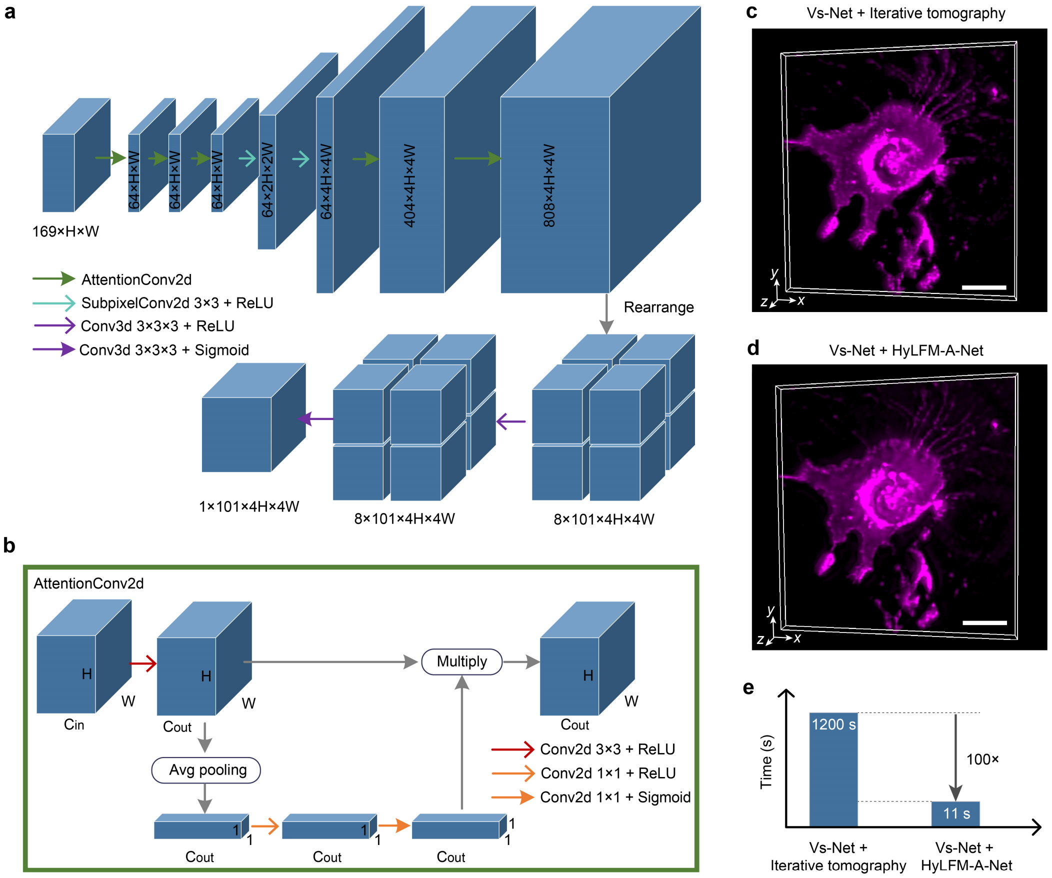

f

HyLFM-Net	feature size			
layer name	c	z[px]	y[px]	x[px]
input	169	1	40	40
Res_2d	808	1	40	40
Res_2d	808	1	40	40
Up_2d	404	1	80	80
Res_2d	404	1	80	80
Proj_2d	707	1	80	80
Rearrange	7	101	80	80
Res_3d	7	101	80	80
Up_3d	7	101	160	160
Res_3d	7	101	160	160
Res_3d	7	101	160	160
Proj_3d	1	101	160	160
Interpolate	1	101	520	520
(Affine)	1	1	520	520

HyLFM-A-Net	feature size			
layer name	c	z[px]	y[px]	x[px]
Vs-output	169	1	120	120
AttentionConv2d	64	1	120	120
AttentionConv2d	64	1	120	120
AttentionConv2d	64	1	120	120
SubpixelConv2d	64	1	240	240
SubpixelConv2d	64	1	480	480
AttentionConv2d	404	1	480	480
AttentionConv2d	808	1	480	480
Rearrange	8	101	480	480
Conv3d	8	101	480	480
Conv3d	1	101	480	480
Interpolate	1	101	520	520

Example:
Input size: 169×40×40
Output size: 101×520×520
Affine layer of HyLFM-Net is used
when trained with light-sheet continuous supervision.
Affine layer of HyLFM-Net is removed
when trained with sLFM reconstruction.
HyLFM-A-Net keeps the Rearrange layer
to transform channel into depth,
but uses attention convolution to extract features.

Supplementary Figure 17 | Attention-based HyLFM-A-Net developed for VsLFM to accelerate the 3D reconstruction process. a, Basic architecture of HyLFM-A-Net, with channel attention applied on 2D convolutions to enhance relevant features for reconstruction. Detailed network descriptions can refer to Methods section. **b**, Unfolded flowchart of the attention convolution. We adopted the squeeze-and-excitation structure, and the convoluted output feature was further multiplied by channel-wise weights with a range from 0 to 1. **c-d,** The similar reconstruction performance of iterative tomography (**c**) and HyLFM-A-Net (**d**) with the Vs-Net output. **e**, Bar plot of the processing time of two methods. The reconstruction time of HyLFM-A-Net is two orders of magnitude less than that of iterative tomography. **f**, Comparison on network architecture and image dimensions between HyLFM-Net and HyLFM-A-Net. Scale bars, 20 μm (**c**, **d**).

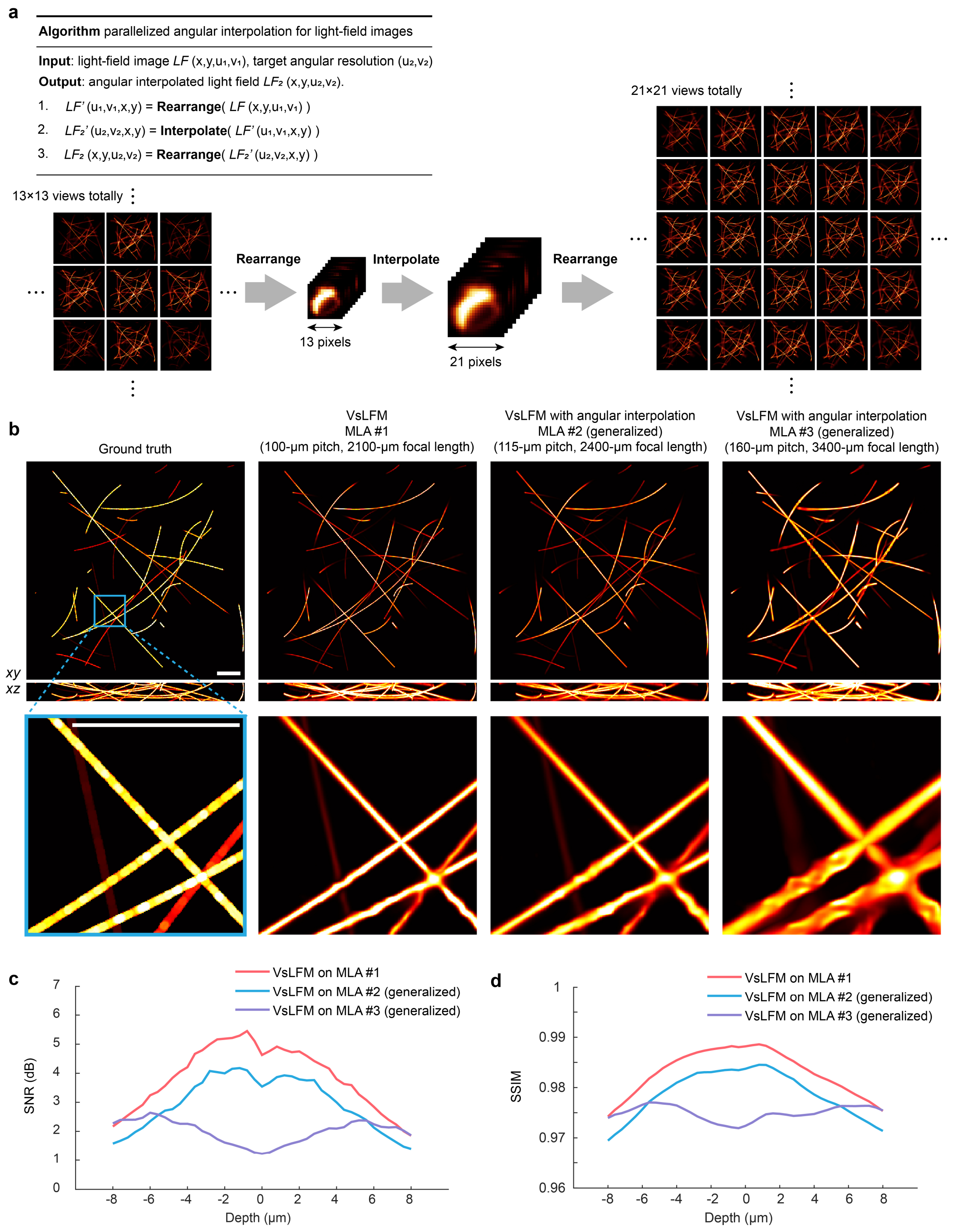

Supplementary Figure 18 | Generalization test of VsLFM to different MLAs. a, Pseudo codes (top) and illustration (bottom) for angular interpolation algorithm. In this illustration, the pixel numbers behind each microlens are set to 13×13 initially and 21×21 after angular interpolation. **b**, Reconstructed orthogonal MIPs of synthetic tubulins with the diameter of 1 μm, acquired by VsLFM with the same Vs-Net model using different microlens arrays (MLAs). The Vs-Net model was pretrained on simulated dataset captured by the system with MLA #1 (100-μm pitch size and 2100-μm focal length). Tubulins captured using MLA #2 (115-μm pitch size and 2400-μm focal length) and MLA #3 (160-μm pitch size and 3400-μm focal length), were firstly proceeded by angular interpolation, and then enhanced by the pretrained Vs-Net model. The synthetic volume serves as

ground truth (left). **c**-**d**, The curves of reconstructed SNR (**c**) and SSIM (**d**) versus different depths by VsLFM with different MLAs. The results indicate that VsLFM with angular interpolation can only adapt to different kinds of MLA with small changes, while MLA with a large change of parameters needs to retrain the Vs-Net model as guided in Methods. Scale bars, 20 μm (**b**).

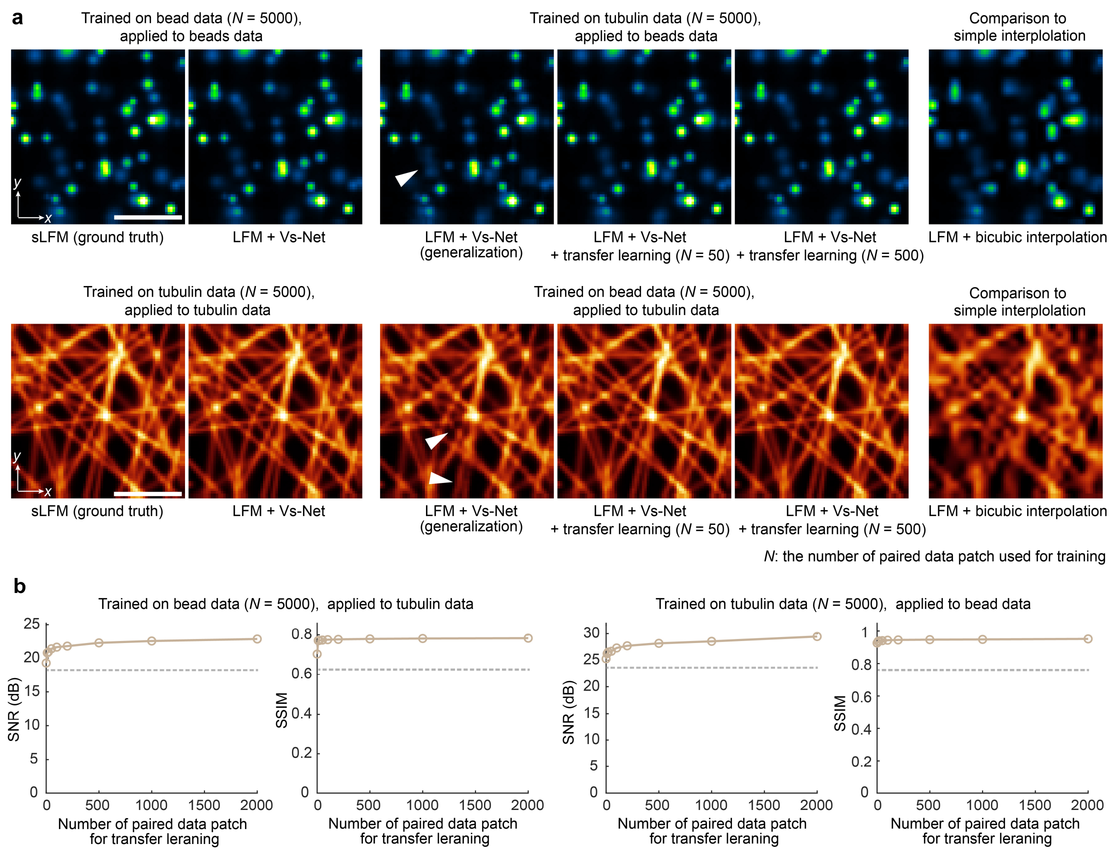

Supplementary Figure 19 | Transfer learning can further improve the performance of Vs-Net. **a**, Cross-sample applications to synthetic beads with the random diameter of 0.5 to 1 μm and synthetic tubulins with the diameter of 1 μm, by Vs-Net models that are solely pre-trained on beads or tubulins, respectively. The two types of samples have large morphological differences, which poses a big challenge on network generalization. Fortunately, Vs-Net with a physics-based learning framework focusing on inherent correlation and disparity of spatial-angular views, generalizes well on different sample types. Transfer learning further makes the network more tractable with a small amount (~1%) of additional data induced, which consequently close the small gap between different sample types. The center views obtained by different methods are shown for comparisons. *N* represents the number of paired data patch used in training process. The results upsampled by direct bicubic interpolation are also shown in the rightmost column for comparisons. **b**, The curves of SNR and SSIM versus the gradually increasing amount of additional data introduced in transfer learning. After pre-trained on one type of data, the network was fine-tuned by transfer learning with increasing amounts (0, 0.2%, 0.4%, 1%, 2%, 4%, 10%, 20%, 40%) of another type of data added, to improve the performance for the latter type of angular views. We used sLFM results as ground truth. Both SNR and SSIM indices were calculated on all 13 × 13 angular views. The gray dotted lines mark the 80% level of the converged value. Scale bars, 10 μm.

Supplementary Table 1 | Detailed network parameters of the Vs-Net

Module and sub-module		Structure	Function
Feature extractor		Spatial-angular Feature Extractor	nn.Conv2d (1,64,3,1,0,1)
		Light field Feature Extractor	nn.Conv2d (1,64, 3,1,13,13)
		Angular-mixed Feature Extractor	nn.Conv2d (1,64,13, 13,0,0)
Interaction and fusion	Spatial-angular interaction module	Convolution	nn.Conv2d (64,64,3,1,0,1)
		Realignment	From light-field domain to spatial-angular domain
		Convolution	nn.Conv2d (64,64,3,1,0,1)
		Concatenation	cat(1)
		Convolution	nn.Conv2d (2*64,64,3,1,0,1)
		Leaky ReLU	nn.LeakyReLU
	K cascade	K cascade	-
	Concatenation	Concatenation	cat(1)
	Fusion	Convolution	nn.Conv2d (K*64,64,3,1,0,1)
		Leaky ReLU	nn.LeakyReLU
		Realignment	From spatial-angular domain to light-field domain
	Light field interaction module	Convolution	nn.Conv2d (64,64, 3,1,13,13)
		Convolution	nn.Conv2d (64,64*13*13,1,1,0,0)
			PixelShuffle(13)
		Realignment	From spatial-angular domain to light-

			field domain
		Convolution	nn.Conv2d (64,64, 3,1,13,13)
		Concatenation	cat(1)
		Convolution	nn.Conv2d (3*64,64, 3,1,13,13)
		Leaky ReLU	nn.LeakyReLU
	K cascade	K cascade	-
	Concatenation	Concatenation	cat(1)
	Angular-mixed interaction module	Convolution	nn.Conv2d (64,64, 1,1,0,0)
		Convolution	nn.Conv2d (64,64,13,13,0,0)
		Leaky ReLU	nn.LeakyReLU
		Concatenation	cat(1)
		Convolution	nn.Conv2d (2*64,64, 1,1,0,0)
		Leaky ReLU	nn.LeakyReLU
	K cascade	K cascade	-
	Concatenation	Concatenation	cat(1)
	Fusion	Convolution	nn.Conv2d (K*64,64, 1,1,0,0)
		Leaky ReLU	nn.LeakyReLU
		Convolution	nn.Conv2d (64,64*13*13,1,1,0,0)
			PixelShuffle(13)
	Final fusion	Convolution	Conv ((K+2)*64,64, 3,1,13,13)
		Leaky ReLU	nn.LeakyReLU
Upsampling		Realignment	From light-field domain to spatial-angular domain

	Convolution	nn.Conv2d (64,64*3*3,3,1,0,1)
	Pixel Shuffle	PixelShuffle(3)
	Convolution	nn.Conv2d (64,1,1,1,0,0)
Global residual	Interpolation	F.interpolate(Bicubic, scale=3)

Notes

nn.Conv2d (input_channel, output_channel, kernel_size, stride, dilation, padding)

cat (concatenation_dimension)

nn.LeakyReLU (negative_slope=0.1, inplace=True)

K: The cascade number of the feature interaction modules

Supplementary Table 2 | Imaging conditions for all fluorescence experiments

	Sample, (imaging T, °C)	Fluorescent label	Exposure time (# time pts)	λ: Power (mW/mm^2)	Volume rate (VPS)	Objective
2a, S4, S5	L929 cells 37 °C	TSPAN4-mCherry	100 ms 1 pts	561: 1.3	-	63×/1.4NA Oil
2b-2c, S7	Fluorescence beads 27 °C	Yellow-green fluorescent (505/515)	100 ms 200 pts	488: 11.7	-	63×/1.4NA Oil
2d	Fluorescence beads 27 °C	Yellow-green fluorescent (505/515)	100 ms 2 pts	488: 11.7	-	63×/1.4NA Oil
4a-4c	L929 cells 37 °C	TOM20-GFP	100 ms 1 pts	488: 0.8	-	63×/1.4NA Oil
4d-4g, S15, SV3	Zebrafish larval 27°C	*Tg(flk:EGFP; gata1: DsRed)*	3 ms 101 pts	488: 5.0 561: 9.0	50	20×/0.5NA Air
5a, SV4	Living mice liver 37 °C	Ly6G (Neutrophil) WGA (vessel)	30 ms 1 pts	561: 3.1 640: 4.2	1.333	63×/1.4NA Oil
5b-5c, SV4	Living mice liver 37 °C	Ly6G (Neutrophil) WGA (vessel)	30 ms 45 pts	561: 3.1 640: 4.2	12	63×/1.4NA Oil
5d, SV4	Living mice liver 37 °C	Ly6G (Neutrophil)	30 ms 141 pts	561: 3.1	0.174	63×/1.4NA Oil

6a-6g, SV6	*Drosophila* 22 °C	*pAce* voltage indicator	1.9 ms 2500 pts	488: 12.8	500	25×/1.05NA Water
6h-6j, SV6	*Drosophila* 22 °C	*pAce* voltage indicator	1.9 ms 8500 pts	488: 12.8	500	25×/1.05NA Water
S9, SV1	L929 cells 37 °C	TSPAN4-mCherry and TOM20-GFP	300 ms 282 pts	488: 0.4 561: 0.5	0.015	63×/1.4NA Oil
S13, SV2	Zebrafish embryo 27 °C	EGFP	150 ms 5400 pts	488: 3.9	3	63×/1.4NA Oil
S16, SV5	Living mice liver 37 °C	Ly6G (Neutrophil) WGA (vessel)	30 ms 401 pts	561: 3.1 640: 4.2	1.333	63×/1.4NA Oil
S17c-S17d	L929 cells 37 °C	TSPAN4-mCherry	100 ms 1 pts	561: 1.3	-	63×/1.4NA Oil

Supplementary Videos

**Supplementary Video 1**	Long-term observation of living L929 cells to demonstrate the resolution improvements of VsLFM.
**Supplementary Video 2**	Experimental comparisons between LFM, VsLFM and sLFM on complicated 3D membrane dynamics in zebrafish embryos *in vivo*.
**Supplementary Video 3**	Highly dynamic circulating blood flow in a zebrafish larva at 50 VPS to compare the generalization ability of VsLFM, VCD-Net and HyLFM-Net, which were all trained on mouse liver data.
**Supplementary Video 4**	VsLFM eliminates the motion artefacts of sLFM in living mouse livers with extremely high-speed motions during respiration.
**Supplementary Video 5**	Intravital subcellular imaging of neutrophil migration and retraction fiber formation by VsLFM with heart beating accompanying in mouse liver.
**Supplementary Video 6**	3D imaging of voltage activities across the whole brain with subcellular resolution in *Drosophila* at ultrahigh speed of 500 VPS, allowing the observation of 3D propagation of action potentials in a single neuron.
