# Supplementary material for: Virtual-scanning light-field microscopy for robust snapshot high-resolution volumetric imaging
Source: Nat Methods. 2023 Apr 6;20(5):735–46. doi: 10.1038/s41592-023-01839-6 (PMC10172145; doi:10.1038/s41592-023-01839-6)
Supplement: Supplementary file 2 — Reporting Summary [file 41592_2023_1839_MOESM2_ESM.pdf]

nature portfolio

Corresponding author(s): Jiamin Wu, Jingyu Yang, Qionghai Dai

Last updated by author(s): Feb 9, 2023

Reporting Summary

Nature Portfolio wishes to improve the reproducibility of the work that we publish. This form provides structure for consistency and transparency in reporting. For further information on Nature Portfolio policies, see our Editorial Policies and the Editorial Policy Checklist.

Statistics

For all statistical analyses, confirm that the following items are present in the figure legend, table legend, main text, or Methods section.

n/a	Confirmed	
☐	☒	The exact sample size (*n*) for each experimental group/condition, given as a discrete number and unit of measurement
☐	☒	A statement on whether measurements were taken from distinct samples or whether the same sample was measured repeatedly
☐	☒	The statistical test(s) used AND whether they are one- or two-sided<br>*Only common tests should be described solely by name; describe more complex techniques in the Methods section.*
☒	☐	A description of all covariates tested
☒	☐	A description of any assumptions or corrections, such as tests of normality and adjustment for multiple comparisons
☐	☒	A full description of the statistical parameters including central tendency (e.g. means) or other basic estimates (e.g. regression coefficient) AND variation (e.g. standard deviation) or associated estimates of uncertainty (e.g. confidence intervals)
☐	☒	For null hypothesis testing, the test statistic (e.g. *F*, *t*, *r*) with confidence intervals, effect sizes, degrees of freedom and *P* value noted<br>*Give P values as exact values whenever suitable.*
☒	☐	For Bayesian analysis, information on the choice of priors and Markov chain Monte Carlo settings
☒	☐	For hierarchical and complex designs, identification of the appropriate level for tests and full reporting of outcomes
☐	☒	Estimates of effect sizes (e.g. Cohen's *d*, Pearson's *r*), indicating how they were calculated

Our web collection on statistics for biologists contains articles on many of the points above.

Software and code

Policy information about availability of computer code

Data collection	LFM and sLFM imaging data were acquired using LABVIEW (2019 version) and our previously published acquisition software, termed sLFdriver (version 2.0, refer to Lu, Z. et al. Nat. Protoc. 2022. https://doi.org/10.1038/s41596-022-00703-9). All relevant codes of VsLFM are readily accessible and available on Github (https://github.com/THU-IBCS/VsLFM-master) and Zenodo (https://doi.org/10.5281/zenodo.7436082)
Data analysis	All data processing and analyses were performed with customized MATLAB (MathWorks, MATLAB 2018b) scripts and Python (3.7 version) scripts. The 3D volumes of Drosophila brain in Fig. 6 were rendered using Imaris (Imaris 9.0.1 software). The 3D rendering of the volumes in supplementary videos was carried out by Voltex modules in Amira (Thermo Fisher Scientific, Amira 2019). The 3D tracking of blood cells in the vessels of the zebrafish larvae and one neutrophil in the vessels of mouse liver were carried out automatically using Imaris.

For manuscripts utilizing custom algorithms or software that are central to the research but not yet described in published literature, software must be made available to editors and reviewers. We strongly encourage code deposition in a community repository (e.g. GitHub). See the Nature Portfolio guidelines for submitting code & software for further information.

Data

Policy information about availability of data

All manuscripts must include a data availability statement. This statement should provide the following information, where applicable:

- Accession codes, unique identifiers, or web links for publicly available datasets
- A description of any restrictions on data availability
- For clinical datasets or third party data, please ensure that the statement adheres to our policy

The Bio-LFSR dataset includes more than 1300 pairs of 4D low-and-high resolution images, covering four species, six structures and multiple imaging conditions, and are made publicly accessible on Zenodo (https://doi.org/10.5281/zenodo.7233421). Supporting data for Vs-Net has been made publicly available on GitHub (https://github.com/THU-IBCS/VsLFM-master/tree/main/Data).

Human research participants

Policy information about studies involving human research participants and Sex and Gender in Research.

Reporting on sex and gender	No human research participants in this study.
Population characteristics	Not involved in this study
Recruitment	Not involved in this study
Ethics oversight	Not involved in this study

Note that full information on the approval of the study protocol must also be provided in the manuscript.

Field-specific reporting

Please select the one below that is the best fit for your research. If you are not sure, read the appropriate sections before making your selection.

☒ Life sciences ☐ Behavioural & social sciences ☐ Ecological, evolutionary & environmental sciences

For a reference copy of the document with all sections, see nature.com/documents/nr-reporting-summary-flat.pdf

Life sciences study design

All studies must disclose on these points even when the disclosure is negative.

Sample size	The sample size is labeled in the figure legends. For the statistics of SBR and resolution characterization experiments, n is mainly determined by the concentration of fluorescent beads. The experiment used a relatively suitable concentration, to prevent the beads from covering each other. For resolution characterization in Fig. 2b, 10 beads with the highest fitting degrees (n =10) per plane and 8 plane in total were used. For Pearson correlation analysis demonstrated in Fig. 3c, n = 17 for each method, which represents by the number of randomly synthetic data. For another Pearson correlation analysis in Supplementary Fig. 14, n = 20 for each method, which was determined by the number of synthetic data. For 3D tracking of blood cells demonstrated in Supplementary Fig. 15, n (= 76) is determined by the specific number of circulating cells at that time. For spike analysis in Figs. 6f and 6g, n = 26 for VsLFM and n = 5 for sLFM, which represent the numbers of identified spikes.
Data exclusions	No data were excluded for the analysis.
Replication	Biological data shown in Figs. 2, 4, 5, 6 and Supplementary Figs. 4, 5, 6, 15, 16 are representative of n = 6 experiments. Biological data shown in Supplementary Figs. 9, 13 are representative of n = 12 experiments. Simulated data shown in Fig. 3 and Supplementary Figs. 10, 12 are representative of n = 17 experiments. Simulated data shown in Supplementary Fig. 1, 8, 11, 14, 17, 18, 19 are representative of n = 20 experiments.
Randomization	Randomization was not relevant to this study, since no experimental group was formed.
Blinding	Blinding was not relevant to this study, since no group allocation was performed.

Reporting for specific materials, systems and methods

We require information from authors about some types of materials, experimental systems and methods used in many studies. Here, indicate whether each material, system or method listed is relevant to your study. If you are not sure if a list item applies to your research, read the appropriate section before selecting a response.

Materials & experimental systems

n/a	Involved in the study
☐	☒ Antibodies
☐	☒ Eukaryotic cell lines
☒	☐ Palaeontology and archaeology
☐	☒ Animals and other organisms
☒	☐ Clinical data
☒	☐ Dual use research of concern

Methods

n/a	Involved in the study
☒	☐ ChIP-seq
☒	☐ Flow cytometry
☒	☐ MRI-based neuroimaging

Antibodies

Antibodies used	Ly6G/Ly6C monoclonal antibody
Validation	The Ly6G/Ly6C monoclonal antibody was validated from the website (https://www.thermofisher.cn/cn/en/antibody/product/Ly-6G-Ly-6C-Antibody-clone-RB6-8C5-Monoclonal/A14748), and purchased to perform mouse experiments.

Eukaryotic cell lines

Policy information about cell lines and Sex and Gender in Research

Cell line source(s)	Mouse L929 (ATCC)
Authentication	The cell lines were not authenticated.
Mycoplasma contamination	The cell line were not tested for mycoplasma contamination.
Commonly misidentified lines (See ICLAC register)	No cell lines used in this study were found in the database of commonly misidentified cell lines that is maintained by ICLAC and NCBI Biosample.

Animals and other research organisms

Policy information about studies involving animals; ARRIVE guidelines recommended for reporting animal research, and Sex and Gender in Research

Laboratory animals	mice (C57BL6/J, ~7-8 weeks), zebrafish (tspan4a mutant), Drosophila (MB065B-GAL4>20×UAS-pAce)
Wild animals	Not involved in this study
Reporting on sex	The mice used in this project are male. The biological sex of zebrafish used in the study is unknown. The Drosophila used in this paper are female.
Field-collected samples	Not involved in this study
Ethics oversight	Animal protocol procedures were reviewed and approved by the Institutional Animal Care and Use Committee office of Tsinghua University.

Note that full information on the approval of the study protocol must also be provided in the manuscript.
